# Genetic Basis of Maize Resistance to Multiple Insect Pests: Integrated Genome-Wide Comparative Mapping and Candidate Gene Prioritization

**DOI:** 10.3390/genes11060689

**Published:** 2020-06-24

**Authors:** A. Badji, D. B. Kwemoi, L. Machida, D. Okii, N. Mwila, S. Agbahoungba, F. Kumi, A. Ibanda, A. Bararyenya, M. Solemanegy, T. Odong, P. Wasswa, M. Otim, G. Asea, M. Ochwo-Ssemakula, H. Talwana, S. Kyamanywa, P. Rubaihayo

**Affiliations:** 1 Department of Agricultural Production, Makerere Univesity, P.O. Box 7062 Kampala, Uganda; dennisokii@gmail.com (D.O.); mwilanatasha@yahoo.co.uk (N.M.); angeltanzito@gmail.com (A.I.); barastere@gmail.com (A.B.); msolemanegy@gmail.com (M.S.); thomas.l.odong@gmail.com (T.O.); wasswa@caes.mak.ac.ug (P.W.); mknossemakula@gmail.com (M.O.-S.); haltalwana@gmail.com (H.T.); Skyamanywa@gmail.com (S.K.); prubaihayo@gmail.com (P.R.); 2 Cereals Program, National Crop Resource Research Institute, P.O. Box 7084 Kampala, Uganda; kdbomet@gmail.com (D.B.K.); otim_michael@yahoo.com (M.O.); grasea9@gmail.com (G.A.); 3 Alliance Bioversity International-CIAT, P.O. Box 24384 Kampala, Uganda; lewismachida@yahoo.co.uk; 4 Laboratory of Applied Ecology, University of Abomey-Calavi, 01BP 526 Cotonou, Benin; agbahoungbasymphorien@gmail.com; 5 Department of Crop Science, University of Cape Coast, P.O. Box 5007 PMB Cape Coast, Ghana; frankkumifk@gmail.com

**Keywords:** combined insect resistance, QTNs, functional prioritization, fall armyworm, maize weevil, stem borers

## Abstract

Several species of herbivores feed on maize in field and storage setups, making the development of multiple insect resistance a critical breeding target. In this study, an association mapping panel of 341 tropical maize lines was evaluated in three field environments for resistance to fall armyworm (FAW), whilst bulked grains were subjected to a maize weevil (MW) bioassay and genotyped with Diversity Array Technology’s single nucleotide polymorphisms (SNPs) markers. A multi-locus genome-wide association study (GWAS) revealed 62 quantitative trait nucleotides (QTNs) associated with FAW and MW resistance traits on all 10 maize chromosomes, of which, 47 and 31 were discovered at stringent Bonferroni genome-wide significance levels of 0.05 and 0.01, respectively, and located within or close to multiple insect resistance genomic regions (MIRGRs) concerning FAW, SB, and MW. Sixteen QTNs influenced multiple traits, of which, six were associated with resistance to both FAW and MW, suggesting a pleiotropic genetic control. Functional prioritization of candidate genes (CGs) located within 10–30 kb of the QTNs revealed 64 putative GWAS-based CGs (GbCGs) showing evidence of involvement in plant defense mechanisms. Only one GbCG was associated with each of the five of the six combined resistance QTNs, thus reinforcing the pleiotropy hypothesis. In addition, through in silico co-functional network inferences, an additional 107 network-based CGs (NbCGs), biologically connected to the 64 GbCGs, and differentially expressed under biotic or abiotic stress, were revealed within MIRGRs. The provided multiple insect resistance physical map should contribute to the development of combined insect resistance in maize.

## 1. Introduction

Despite the importance of maize (*Zea mays* L.) for food security, income, livestock feed, and biofuel products, and its large production area, grain yield remains low in sub-Saharan African (SSA) countries, averaging less than 1.8 ton/ha due to a barrage of biotic and abiotic stresses [1]. Maize faces several yield-limiting factors, among which biotic stresses such as insect pest attacks [1,2,3] start in the field with a range of voracious phytophagous pests that include stem borers, leaf feeders, phloem feeders, and root feeders [4]. In SSA, field and storage pests cause estimated yield losses ranging from 10% to 90% of the seasonal production [5,6]. Recently, fall armyworm (FAW), *Spodoptera frugiperda* Smith (Lepidoptera, Noctuidae), migrated to Africa through West and Central African countries [7] and has since spread throughout the continent [8]. The pest is now a threat to food security in Africa owing to its voracious and polyphagous nature, resulting in substantial yield losses in maize production [1]. Besides, storage pests (SP) such as the maize weevil (MW), *Sitophilus zeamais* Motsch (Coleoptera: Curculionidae), and the larger grain borer (LGB), *Prostephanus truncatus* Horn (Coleoptera: Bostrichidae), have a substantial share in these losses, especially in Africa where poor storage facilities expose stored grains [5,9].

Chemical control measures are widely used to reduce maize yield losses incurred from attacks by field insect pests, and MW and LGB on stored grains. However, insecticides, although efficient in reducing insect pressure, pose a significant health hazard to maize consumers and are harmful to the environment [1,10]. Furthermore, pesticides are unaffordable to small-scale farmers in Africa and may result in the development of chemical resistance in insects, and the emergence of secondary pests. Further, the application of insecticides in the field represents a threat to nontarget organisms including natural enemies of insect pests like FAW [1,10,11]. Another control measure is host plant resistance (HPR), which is the inherent plant ability to limit insect damage through various defense mechanisms provided by its genetic make-up [12,13] and is fully compatible with all other intergrate pest management strategies. Host plant resistance at its highest level can be exclusively applied to thwart insect attacks without expensive and controversial interventions. Considering the plethora of insect species that either simultaneously or concurrently attack all maize parts, including leaves, stems, and kernels [4], the development of HPR should target multiple insect resistance [14].

Understanding the genetic basis of multiple insect resistance is critical to the control of combinatorial attacks from field and storage insect pests which are critical constraints to maize productivity and storability, especially in sub-Saharan Africa, causing both high yield and grain quality losses through damage and mycotoxin contaminations. However, most genetic, biochemical, and genomics studies on plant resistance mechanaisms were directed towards understanding maize resistance mechanisms to single insect pests [15]. Using the single insect paradigm, several quantitative trait loci (QTL) for maize resistance to insects were discovered for FAW, stem borers (SB), and SP. These were meta-analyzed in a previous study to better understand the genetic basis of maize resistance to multiple insect pests and explore avenues of multiple insect resistance breeding [16]. However, there was a paucity of African germplasm in these meta-analyses since very few quantitative trait loci (QTL) mapping studies were conducted for SB [17] and SP [18], and no study had been carried out for maize resistance to FAW. Therefore, the meta-QTL (MQTL) information resulting from these meta-analyses can not be confidently used in African breeding programs targeted at developing maize varieties resistant to multiple insect pests. The challenges encountered in the extrapolation of these results to African backgrounds also stem from the co-evolutionary basis of the maize–insect interaction characterized by a concomitant development and deployment of plant defense and insect counter attack mechanisms that could substantially vary from one background to another [19,20,21].

Currently, genome-wide association studies (GWASs) constitute the most advanced strategy for mapping regions of the genome of a species that are associated with a phenotype or a set of traits of interest to plant and animal breeders and geneticists [22]. Compared with biparental QTL analyses, they take advantage of the high diversity and multiple recombination history that is available in natural populations to narrow down QTL resolution to the nucleotide level (i.e., quantitative trait nucleotide (QTN)) and allow increased statistical power [23]. In maize, a GWAS was used to map several complex traits including disease and insect resistance, for example, resistance to maize chlorotic mottle virus and maize lethal [24] and response to the Mediterranean corn borer (MCB) [25,26,27]. However, to the best of our knowledge, no GWAS was reported on Africa-adapted maize germplasms for their response to locally occurring insect pests such as FAW and MW.

A logical follow-up to mapping studies is the identification of promising candidate genes (CGs) around the QTNs associated with the traits of interest to help interpret their biological significance [28]. However, not all genes neighboring a QTN are functionally associated with the regulation of the traits in consideration, and often, the genes could be numerous, therefore, requiring filtering to come up with a list of high-confidence CGs [29]. A prioritization of the CGs identified within a defined window containing QTNs is necessary to avoid expensive validation experiments of numerous potentially unfit genes [30]. In maize, the prioritization of CGs based only on the available genetic information related to the genes is limited, since only 1% of the maize genome is annotated [31,32]. Therefore, in prioritizing CGs, integrative approaches involving ontology-based semantic data integration with expression profiling, comparative genomics, phylogenomics, functional gene enrichment, and gene network inference analyses represent a promising alternative [30,33,34]. Such an approach would take advantage of the extensive genomic information available in maize and its sister species such as rice (*Oryza sativa*) and its more extant relative *Arabidopsis thaliana* [35,36,37].

Therefore, in this study, we conducted a GWAS to identify QTNs for resistance to either FAW or MW or both insect pests in a diverse association mapping panel (AMP) composed of a genetically diverse set of maize inbred and doubled haploid (DH) lines developed in a wide range of African agro-ecologies. Such diverse populations are suitable for GWAS analyses on traits such as insect resistance, owing to the high genetic diversity and rapid linkage disequilibrium (LD) decay that characterizes tropical maize germplasm [38]. Further, the GWAS results were compared with those of the QTL meta-analysis conducted earlier [16] to assess the consistencies of the positions of the insect resistance-associated genomic regions. Furthermore, to establish a list of promising CGs for insect resistance that could be incorporated in molecular breeding programs, a suite of functional genomics approaches were used to identify, functionally characterize, and prioritize genes located in the vicinity of markers and genomic regions associated with maize resistance to insect pests.

## 2. Material and Methods

### 2.1. Association Mapping Panel (AMP) Establishment and Field Planting

The AMP used in this study consisted of 358 maize lines from a diverse genetic and geographic background sourced from the National Crop Resources Research Institute (NaCRRI of Namulonge, Uganda), the International Institute for Tropical Agriculture (IITA of Ibadan, Nigeria), and The International Maize and Wheat Improvement Center (CIMMYT of Nairobi, Kenya). The AMP was composed of 71 inbred lines developed for various purposes at NaCRRI, 5 stem borer-resistant inbred lines from IITA, 28 stem borer (SB)-resistant lines [39,40], 19 storage pest (SP)-resistant inbred lines [41,42], and 4 doubled haploid (DH) populations of 235 lines developed from insect-resistant parents at CIMMYT. The DH lines from CIMMYT were developed from six parents of which three were stem borer-resistant and one was a storage pest-resistant inbred line (these were also included in the AMP), and two were CIMMYT maize lines ( CML) elite lines (one, CML132, was included in the AMP) (Table S1).

The AMP was planted in an augmented design in three environments at the Mubuku Irrigation Experimental Station in Kasese (316 lines including six replicated in 12 blocks) during the second rainy season (2017B) and at NaCRRI of Namulonge in 2018 (92 lines including two checks replicated in five blocks) and 2019 (252 lines including four checks replicated in 10 blocks), both during the first rainy seasons (2018A and 2019A, respectively). Each combination of location and season was considered an environment amounting to three environments.

The Mubuku Irrigation Experimental Station is located in Kasese, western Uganda (0°16′10″ N, 30°6′9″ E; 1330 m asl), and receives 1000 mm of rainfall annually. The soils are characterized as sandy loam soils with a pH of 5.68, just above the 5.5 critical pH level for common bean production. The National Crop Resources Research Institute (NaCRRI) Namulonge is located in central Uganda (0°31′30″ N, 32°36′54″ E; 1160 m asl). Namulonge receives 1300 mm of rainfall annually, and soils are characterized as oxisols with a pH of 5.8 [43]. 

### 2.2. Genotyping and Quality Control and Assurance of SNP Markers

Maize leaves at the sixth-leaf stage of development were harvested from 5–10 plants per plot in 2017B and completed in 2018A (for lines not captured in 2017B), oven-dried overnight at 35 degrees Celsius and shipped to Biosciences east and central Africa (BecA) of the International Livestock Research Institute (ILRI of Nairobi, Kenya) for genotyping. Diversity Array Technology (DArT) genotyping facilities [44] were used to identify 34,509 SNPs from 341 lines of the AMP. For quality assurance of the genetic data prior to further genomic analyses, duplicate SNPs were first removed using the R package DartR [45] to remain with 28,919 unique SNPs (DRSNP). To reduce the negative effect of GWAS multiple-testing on the association discovery statistical power, the 28,919 DRSNPs were pruned based on linkage disequilibrium (LD) among the SNPs (*r*^2^ = 0.2 and window size = 500,000 bp). This operation was performed using the R package SNPRelate [46] and allowed to reduce the number of SNPs considered for GWAS to 3124 SNPs in LD (LDPSNPs), spanning the whole maize genome with a fairly even marker distribution (Figure S1). The 3124 LDPSNPs were then imputed in TASSEL 5 with the LD KNNi imputation method [47].

### 2.3. FAW Damage Scoring and MW Bioassay

After germination, plants were left unprotected to allow sufficient natural pressure from the FAW population. FAW damage scoring in all three environments was carried out two months after planting based on a visual assessment on a scale of 1 (no or minor leaf damage) to 9 (all leaves highly damaged) as described by [48] and illustrated in Figure S2 [49].

The rearing and bioassay for MW was performed as described in previous experiments carried out at NaCRRI [50,51]. Weevils were reared prior to the bioassay to obtain enough insects aged between 0 and 7 days for infestation. During rearing, standard conditions were provided to weevils to ensure proper acclimatization during the experiment. Rearing was carried out by creating a weevil–maize grain culture of 300 to 400 unsexed insects and 1.5 Kg of grains contained in 3000 cm^3^ plastic jars incubated for 14 days in the laboratory at a temperature of 28 ± 2 °C and relative humidity of 70 ± 5%, to enhance oviposition. The lids of the jars were perforated and a gauze-wire mesh of a pore size smaller than 1 mm was fitted in each of the lids to allow proper ventilation while preventing the weevils from escaping.

After harvest and shelling, 30 g of grains from each line of the AMP were weighed from a bulk of all three environments with the aim of having three replicates per genotype. Due to the limited seed quantities, 64 lines were replicated thrice, 123 lines were replicated twice, and 132 once. Each of these samples was wrapped in polythene bags and kept at −20 °C for 14 days to eliminate any weevil infestation prior to the start of the experiment. After this disinfestation process, samples were left to thaw and were transferred into 250 cm^3^ glass jars and infested with 32 unsexed weevils. After the 10-days incubation to allow oviposition, all dead and living adult insects were removed. One month after infestation (MAI), each sample was removed from its jar, and the grains and the flour were separated and their weights were recorded. The total number of holes inflicted by the weevils on the grains were counted along with the number of holed grains. Further, the number of dead and living weevils was recorded. After these measurements were collected, the grains were returned to their respective jars and all the measurements were repeated at 2 and 3 MAI. The collected data were used to infer, for each sample, the cumulative grain weight loss (GWL), the cumulative number of emerged adult weevil progenies (AP), the cumulative number of damage-affected kernels (AK), the cumulative number of holes on grains (NH), and the cumulative weight of the flour produced (FP).

### 2.4. Statistical Analyses of the Phenotypic Data

An analysis of variance (ANOVA) was performed using the package lme4 [52] implemented in the R environment [53] to determine genetic variability among the lines of the AMP for the MW and FAW resistance traits. The linear model for MW traits (GWL, AP, AK, NH, and FP) was as follows:

(1) Y=μ+Replication+Genotype+Error

The models for FAW damage scores for FAW in single and across environments were as follows:

—FAW individual environments 2017B and 2019A:

(2) Y=μ+Block+Genotype+Error

—FAW individual environment 2018A:

(3) Y=μ+Replication+Block+Genotype+Error

The FAW across environments model was:

(4) Y=μ+Location+Block+Genotype+Location:Genotype+Error

where μ is the grand mean of the target trait.

The genotype-based heritabilities (H^2^) for MW and FAW resistance traits were calculated on a genotype mean basis [54] using variance components obtained from a mixed model considering the effects of all the factors present in models 1, 2, 3, and 4 as random, using the following formulas:

For MW resistance traits:

$$H^{2}=\frac{Variance\;Genotypes}{Variance\;Genotypes+\left(Variance\;Error/NR\;\right)}$$

For FAW damage scores in 2017B and 2019A:

$$H^{2}=\frac{Variance\;Genotypes}{Variance\;Genotypes+Variance\;Error}$$

For FAW damage in 2018A:

$$H^{2}=\frac{Variance\;Genotypes}{Variance\;Genotypes+\left(Variance\;Error/NR\right)}$$

For FAW damage scores across environments:

$$H^{2}=\frac{Variance\;Genotypes}{Variance\;Genotypes+\left(\left(Variance\;Genotypes:Enviroments+Variance\;Error\right)/NE\right)}$$

where NE is the number of environments and NR is the number of replications.

Then for the GWAS analyses of maize resistance to MW traits and FAW damage scores across environments, mixed models 1 and 4 were used to extract best linear unbiased predictors (BLUPs) using the package lme4 [52]. Pairwise Pearson correlations among BLUPs of MW and FAW resistance traits were computed and visualized with the R package PerformanceAnalytics (https://cran.r-project.org/web/packages/PerformanceAnalytics/index.html).

### 2.5. Linkage Disequilibrium (LD), Population Structure and Kinship Matrix

The software TASSEL v5.2 [47] was used to calculate LD with the squared Pearson correlation coefficient (r^2^) between pairs of SNPs, and principal components (PCs) and the kinship matrix to infer the population structure and cryptic relatedness with the AMP. The LD decay graph, plotting the *r*^2^ between pairs of SNPs against their pairwise physical distance and showing the average pairwise distances at which LD decayed at *r*^2^ = 0.1 and 0.2, was generated as described earlier [27,55], based on Remington et al. [56]. The kinship matrix was generated using the centered identity by state (Centered-IBS) function. Further, 345 PCs accounting for 100% of the variance explained by the 3124 LDPSNPs were generated.

### 2.6. Genome-Wide Association Mapping

The BLUPs for all traits were transformed using the R package bestNormalize [57] that tests a suite of normalizing transformation methods on the values of each trait and chooses the one that fits best the data based on a goodness of fit statistic. A multi-locus genome-wide association study (GWAS) was conducted for all MW and FAW traits using both transformed and untransformed BLUPs with the 3124 LDPSNPs to allow comparing the results and choosing the best based on the Manhattan and Q–Q plots. The multi-locus GWAS was conducted using the Fixed and random model Circulating Probability Unification (FarmCPU) package [58] implemented in R packages Genome Association and Prediction Integrated Tool (GAPIT) [59] and Memory-Efficient, Visualize-Enhanced, Parallel-Accelerated GWAS Tool (rMVP) (https://github.com/XiaoleiLiuBio/rMVP), to solve the mixed problem of false positive and false negative SNPs usually encountered in the traditional mixed linear model (MLM) [60,61,62]. For that, FarmCPU uses a modified MLM, the multi-loci mixed model (MLMM) to incorporate both the kinship matrix and PCs to account for the varying relatedness and the population stratification present among the lines in the AMP [58]. To control the population structure which can differentially affect traits in an AMP [30,63,64], the number of PCs included in the GWAS models for each trait was gradually increased until the achievement of an adequate control of the false positive and false negative rate through inspection of the Q–Q plot of the observed against the predicted negative log10 (*p*-values) of each of the 3124 LDPSNPs [30,65]. The number of PCs included for the analysis of each trait is presented in Figure S3.

Three Bonferroni genome-wide significance levels (BGSL), 1%, 5%, and 30% (for suggestive associations), were used to identify SNPs significantly associated with resistance traits. Based on the B73 maize genome reference (AGPv4) coordinates, the physical positions of the SNPs significantly associated with any of the resistance traits were compared with those of the MQTL identified in the previous study of Badji et al. [16]. It should be noted that this map was earlier enriched with additional QTL for maize resistance to spotted stem borer (SSB) and African maize stalk borer (AMSB) [17], MW [18] in African backgrounds, the Asian corn borer (ACB) [66], and FAW and southwestern corn borer (SWCB) [67,68,69], and QTLs for the benzoxazinoids content [70,71,72]. Based on the AGPv4 coordinates of QTL and MQTLs, a physical map was generated and visualized using MapChart V2.3 [73].

## 3. Candidate Gene (CG) Designation

### Pre-CGs (Pre-CGs) Identification around the QTNs for Maize Resistance to FAW and MW

Genes containing or located within a 10,000 base pairs (10 Kb) window including the QTNs for single or combined resistance to FAW and MW were recorded as pre-CGs on the B73 maize reference genome (AGPv4) on the Ensembl Plant databases (http://plants.ensembl.org/Zea_mays). This search window was extended to 30 Kb when no gene was discovered within the immediate 10 Kb containing the QTN. The genetic information related to these pre-CGs, namely gene name, description, and AGPv4 coordinates, was retrieved from the maize genome database (https://www.maizegdb.org/) and their canonical protein sequences were downloaded from the same database. The distance that separates each pre-CG from its respective QTN (DQTN) was calculated based on their AGPv4 respective coordinates as the difference between the end position (for genes upstream the QTNs) or start position (for genes downstream the QTNs) and the position of the QTN they were associated with.

## 4. Pre-CGs Prioritization Through a Suite of Functional Characterizations

### 4.1. In-Silico Expression Analyses of the Pre-CGs

To determine whether the expression of any of the pre-CGs are up- or downregulated under certain biotic and abiotic stress conditions, and to reveal in which maize organs and developmental stages they were expressed, an in silico differential gene expression analysis was carried out using the condition search tools, “Perturbation”, “Anatomy”, and “Development”, respectively, of the software GENEVESTIGATOR V7.4.0 (https://genevestigator.com/gv/doc/introplant.jsp) [74]. For these analyses, the maize microarray platform, mRNA-seq Gene Level Zea mays (ref: AGPv4), was used to evaluate the expression levels of the pre-CGs. From this platform, for the “Perturbation”, “Anatomy”, and “Development” analyses, only maize experiments relevant to biotic and abiotic stresses were selected and the in silico experiments were performed separately for each category of stress whilst including in the biotic stress category plant biochemicals reported to have an influence in biotic stress resistance, for instance, jasmonates and jasmonate-like chemicals. The in silico pre-CG differential expression analyses were also conducted using the Gene Expression Atlas (https://www.ebi.ac.uk/gxa/home) [75] and *Zea mays* for maize was chosen as a species. Options “Treatment”, “Growth conditions”, “Biotic plant treatment”, “Stimulus”, “Infect”, and “Environmental stress” were checked and experiments not concerned with biotic or abiotic stress were filtered out.

### 4.2. Identification of Conserved Domains within the Protein Sequences of the Pre-CGs

A protein conserved domain search was performed for the pre-CGs on the national center for biotechnology information (NCBI) conserved domain (CD) database (https://www.ncbi.nlm.nih.gov/Structure/bwrpsb/bwrpsb.cgi) using the CDD\SPARCLE Batch Web CD-Search Tool with default parameters [76].

### 4.3. Identification of Pre-CG Orthologs and Co-Expression Analysis

For functional inferences, *A. thaliana* and rice (*Oryza sativa)* orthologs of the pre-CGs were identified using the ortholog search tool of the web-based database g:Profiler (https://biit.cs.ut.ee/gprofiler) and their gene ids and functional information were retrieved. The protein sequences of the *A. thaliana* genes were downloaded from TAIR (https://www.arabidopsis.org/tools/bulk/sequences/index.jsp). For the rice genes, the gene ids were converted from the RAP to the MSU formats using the Id Converter tool from the Rice Annotation Database (rap-db) (https://rapdb.dna.affrc.go.jp/tools/converter) and their protein sequences were retrieved from the Rice Genome Annotation Project (http://rice.plantbiology.msu.edu/downloads_gad.shtml). A whole-genome co-expression analysis between the pre-CGs and their rice and *A. thaliana* orthologs was conducted using the gene protein sequences tool on the web-based server OrthoVenn 2 (https://orthovenn2.bioinfotoolkits.net) [77].

### 4.4. Pre-CG Prioritization

The functional information obtained either from the pre-CG (functional and conserved protein domains information) or inferred from their co-expressed orthologs (gene functional information) was searched in the scientific literature along with several plant biotic and abiotic stress-related keywords to ascertain the relation with plant defense mechanisms. This information along with the results from the in silico expression analyses were considered as evidence of involvement in plant defense mechanisms. Then, pre-CGs were classified into three categories (CGC): A (more than one evidence), B (only one evidence), and C (no evidence), and those falling within A and B categories were considered as putative GWAS-based CGs (GbCGs).

### 4.5. Network-Assisted CG Discovery for Multiple-Insect Resistance

The putative GbCGs were used to discover other functionally connected genes located within the multiple insect resistance genomic regions (MIRGRs) determined in a previous study [16]. The maize co-functional network database, MaizeNet (http://www.inetbio.org/maizenet/) [78] was used to identify maize genes functionally connected to the GbCGs used as guide genes. The network-based CGs (NbCGs) with connectivity scores to the guide genes higher than 5 were assessed for in silico differential expression using the Gene Expression Atlas (GXE) [75] with the same parameters as described earlier in this paper. The genes up- or downregulated were selected and their genomic locations were checked within the IRGRs to designate them as NbCG for maize resistance to insect pests.

### 4.6. Interactions Among CGs

To investigate the possible interactions among the CGs (GbCGs and the NbCGs) as evidence of their involvement in a network-like defense mechanism, protein–protein interaction analyses were carried out by submitting protein sequences of both CG groups to the STRING v11 database (http://string-db.org/) [79]. Further, pathway functional enrichment analyses were conducted for the CGs using the Ghost tool of the web-based platform g:Profiler β (https://biit.cs.ut.ee/gprofiler/gost#) using a Bonferroni correction threshold of 0.05 [80]. The interaction network of the gene ontology molecular function was visualized using the software EnrichmentMap [81] implemented in the software Cytoscape V3.7.2 [80].

## 5. Results

### 5.1. Trait Variance, Heritability, and Correlations

There was a highly significant (*p* < 0.001) genetic variation among the lines of the AMP for FAW and all MW resistance traits collected and analyzed in this study, except FAW damage in 2017B which was significant at *p* > 0.01. For FAW resistance traits, the genotypic effect was highly significant in 2018A, 2019A, across environments (*p* < 0.001), and in 2017B (*p* < 0.01). All other factors showed at least a 5% significant difference, except the block effect in 2019A (Table 1).

**Figure 1:**
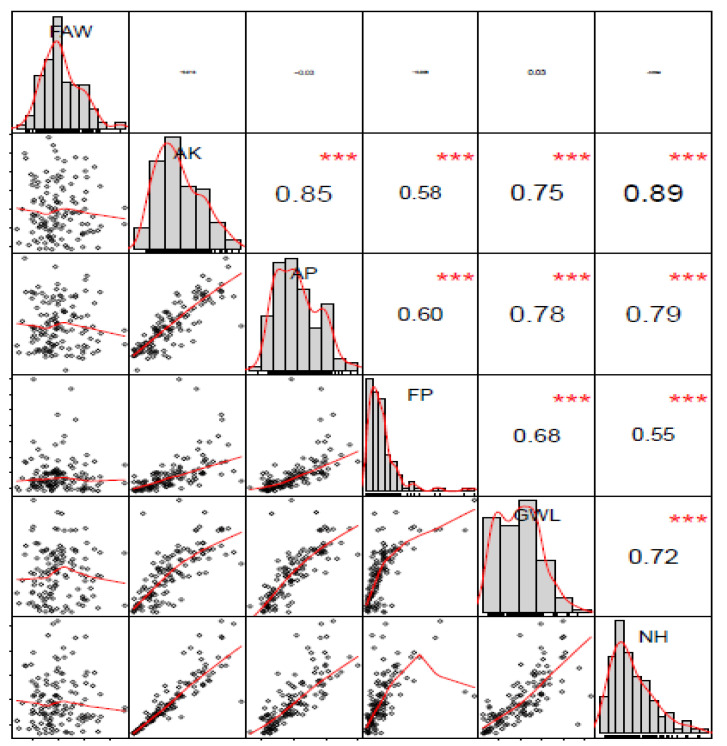
Pairwise Pearson phenotypic correlation among FAW damage and MW traits. AK = number of affected kernels, NH = number grain holes, AP = number of emerged adult progenies, FAW = Fall armyworm, FP = total amount of flour produced, and GWL = grain weight loss.

For MW resistance traits, all genotype effects were highly significant (*p <* 0.001), however, the effect of the replications was only significant (*p* < 0.5) for adult progeny emergence (AP) and the number of holes (NH) (Table 2). The non-signifcant variances attributed to the replication effect is because replicates for the MW bioassay were technical rather that biological. Heritability values for FAW damage scores were high in individual environments, varying from 0.67 in 2018A to 0.80 in 2017B (Table 1). However, the H^2^ for FAW damage scores across environments was relatively low (H^2^ = 0.25) as a result of high significance (*p* < 0.001) of the influence of environmental factors and their interaction with the genotypes (Table 1). Traits related to MW resistance recorded high heritability (H^2^) values ranging from 0.78 for grain weight loss (GWL) to 0.95 for flour production (FP) (Table 2). Pearson correlations (*R*) among MW resistance traits were highly significant while FAW damage was poorly and mostly negatively correlated with MW resistance traits, and all were non-significant (Figure 1).

The *R* values for MW resistance traits ranged from 55% between FP and NH to 89% between AK and NH. The *R* values were the lowest whenever FP was included in a pairwise correlation with other MW resistance traits with R ranging from 55% (FP vs. NH) to 60% (FP vs. AP).

### 5.2. Association Mapping for MW and FAW Resistance Traits

**Figure 2:**
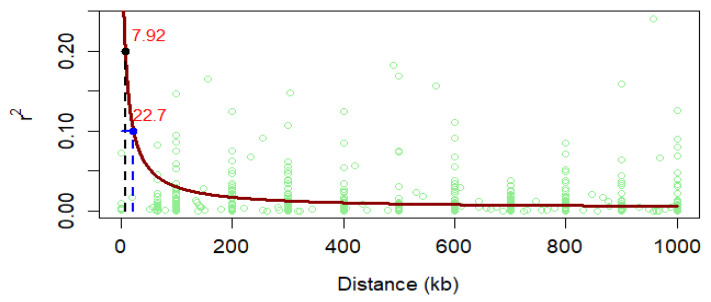
Linkage disequilibrium (LD) plot representing the average genome-wide LD decay in the panels with genome-wide markers. The values on the *y*-axis represent the squared correlation coefficient, r^2^, and the *x*-axis represents the physical distance in kb.

**Figure 3:**
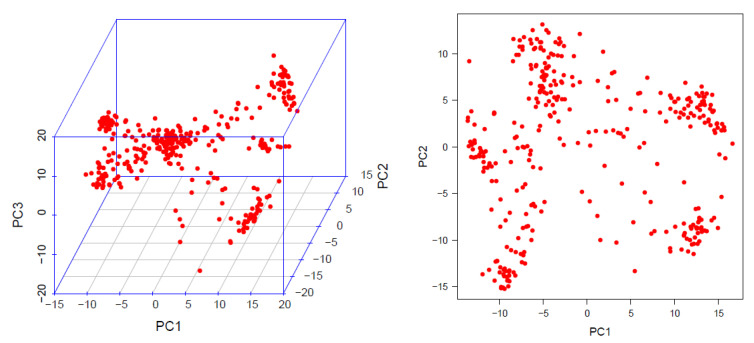
3D and 2D distribution of the maize lines composing the association mapping panel according to the first three principal components (PC1, PC2, and PC3) generated from the 3124 LD-pruned markers.

**Figure 4:**
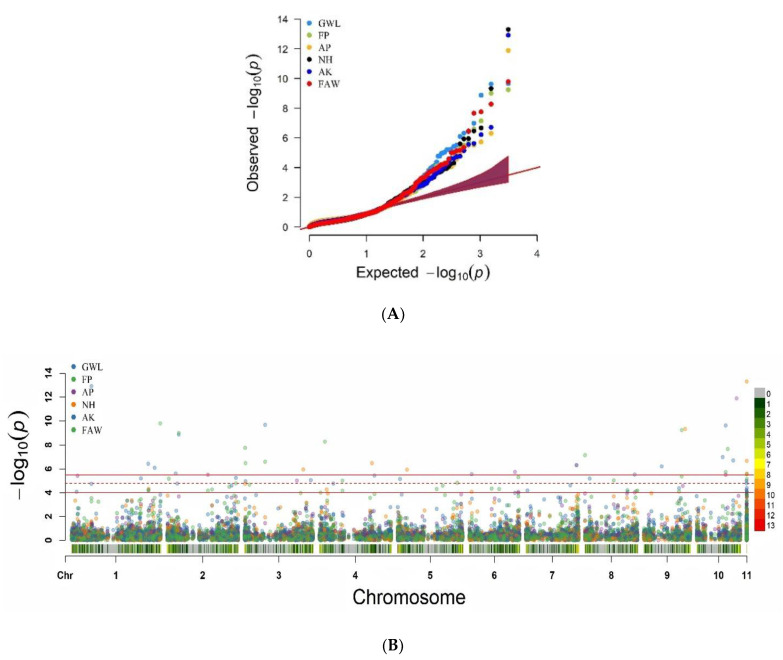
Combined Q–Q (**A**) and Manhattan (**B**) plots derived from the genome-wide association analysis for fall armyworm (FAW) damage and maize weevil (MW) traits. Bonferroni genome-wide significance levels of 0.01 (upper line), 0.05 (middle lines), and 0.3 (lower line) on B. Single- and multiple trait-associated traits.

Linkage Disequilibrium and Effective Control of Population Structure and Kinship. The whole-genome linkage disequilibrium (LD) was computed using the 3124 LD-pruned SNP markers and the genome-wide LD decay plot was generated from the LD (*r*^2^) between the adjacent pair of markers on the *y*-axis and the distance in kb on the *x*-axis (Figure 2). A rapid LD decay characterized the AMP with the average physical distance increasing from 7.92 to 22.7 when the cut-off point decreased from r^2^ = 0.1 to 0.2, which was promising for GWAS and CGs. The association mapping panel (AMP) used in this study was composed of maize lines of diverse origins, suggesting the existence of a population structure as highlighted in Figure 3. However, the relative clustering of these subpopulations was not well defined (no group was clearly separated from the other ones) due to the complex kinship relatedness shared by the majority of the lines. This population structure leads to biased SNP–trait associations if not properly accounted for in this study. Therefore, allocating an adequate number of PCs for each trait (Figure S2) and including a kinship relationship matrix minimized the rate of false positives and negatives as evidenced in Figure 4. The Q–Q plot in Figure 4A shows that the population parameters (kinship and population structure) were effectively controlled for all traits analyzed, hence minimizing the rate of both false positive and false negative associations. Besides, both the original and transformed BLUPs of all the FAW and MW resistance traits were tested in the GWAS analyses. The analysis with transformed BLUPs generated better-looking plots than those with the non-transformed BLUPs for most traits except GWL and FP, for which population parameters could only be successfully controlled using the transformed BLUPs. Several SNP–trait associations or quantitative trait nucleotides (QTNs) were discovered at very stringent Bonferroni genome-wide significance levels (BGSL) of 0.05 and even 0.01 for all the resistance traits analyzed (Figure 4B).

Sixty-two QTNs distributed on all the 10 maize chromosomes were significantly (at least below 0.3 BGSL) associated with either single or both MW and FAW resistance traits, of which, 47 and 31 were significant at 0.05 and 0.01, respectively (Table 3). Chromosomes 6 and 7 did not harbor any QTN associated with FAW damage resistance, whereas all 10 chromosomes were involved in at least one QTN for maize resistance to MW. Of the 62 QTNs, 14 were found to influence the response to FAW (9 QTNs at 0.05), while many other QTNs were associated with resistance to MW traits such as GWL (17 QTNs of which 14 at 0.05), FP (17 QTNs of which 7 at 0.05), AK (13 QTNs of which 6 at 0.05), AP (10 QTNs of which 7 at 0.05), and NH (8 QTNs of which 4 at 0.05). Sixteen QTNs were associated with resistance to multiple traits, of which, six were associated with resistance to both FAW and MW, suggesting possible pleiotropic effects.

The QTNs for multiple-insect resistance are 100024832-19-A/C at position 263,624,976 on chromosome 1 for GWL and FAW, 9714175-54-C/G at position 2,734,515 for FAW and NH and 4764930-10-C/T at position 4,141,348 for FAW and GWL, both located on chromosome 3, 100220678-45-A/G at position 78,882,987 on chromosome 4 for FAW and FP, 2559495-18-T/G at position 146,321,767 on chromosome 8 for FAW and AK, and 9682691-38-C/T at position 129,393,054 on chromosome 9 for FP and FAW.

### 5.3. Resistance-Related QTN-QTL-MQTL Co-Localizations

The QTNs discovered in the current study were projected on a physical map along with MIRGRs previously discovered through meta-analyses [16]. The majority of the QTNs for resistance to single or combined FAW and MW resistance fell within the single and multiple insect resistance MQTL concerning several field pests such as the European corn borer, the Mediterranean corn borer, the sugarcane borer, and the southwestern corn borer and the storage pest MW, along with QTLs for several cell wall constituents. Further, the QTNs co-localize with several QTL for resistance to other insect species such as the Asian corn borer (ACB), the spotted stem borer (SSB), the African maize stall borer (AMSB), FAW, and MW, and QTL for the maize benzoxazinoids content on virtually all the 10 maize chromosomes. The co-localizations of maize biochemical and resistance genetic factors to multiple insect pests formed several clusters on most of the maize chromosomes, especially in the top and bottom chromosomal regions (Figure S4).

### 5.4. Pre-CGs Functionally Associated with Plant Stress Response Mechanisms in the Vicinity of the QTNs

Seventy-eight pre-CGs were identified for 58 QTNs (Table S2). These pre-CGs include transcription factors, protein kinases, disease resistance genes, leucine-rich repeat, and basic leucine zipper genes. Four QTNs did not have a gene located in a window of 30 Kb, namely 4593663-22-G/A on chromosome 3 at 71,004,409 bp for GWL and FP, 4587005-7-C/G on chromosome 6 at 9,188,598 bp for AK and NH and 4579331-18-T/C at 157,597,555 bp for AP, and 4776702-53-G/A on chromosome 10 at 125,628,521 bp for AK (Table S2). Most of the pre-CGs (44 in total) contained the QTNs whilst others were very closely located with the farthest, Zm00001d049175, being at 15,726 bp downstream the QTN 2381322-13-C/G associated with resistance to FAW (Table S2). For the QTNs associated with combined resistance to FAW and MW, five were associated with only one gene each, suggesting the nature of the genetic control as pleiotropy. Only 100024832-19-A/C on chromosome 1 presented two pre-CGs, of which, one (TATA-binding protein1, Zm00001d033472) contains the QTN of interest while Zm00001d033471, a putative DNA-binding protein, was located 3094 bp upstream (Table S2). Several protein conserved domains were found within 62 of the pre-CGs. Twenty-three of these 62 pre-CGs presented protein conserved domains that are functionally associated with plant biotic and abiotic stress defense mechanisms. These features include the WRKY, F-BOX, NAM, bZIP, LRR, AUX_IAA, zf-C2H2, and GTP-binding protein domains (Table S2).

### 5.5. Pre-CGs Differentially Expressed under Biotic and Abiotic Stress Conditions

**Figure 5:**
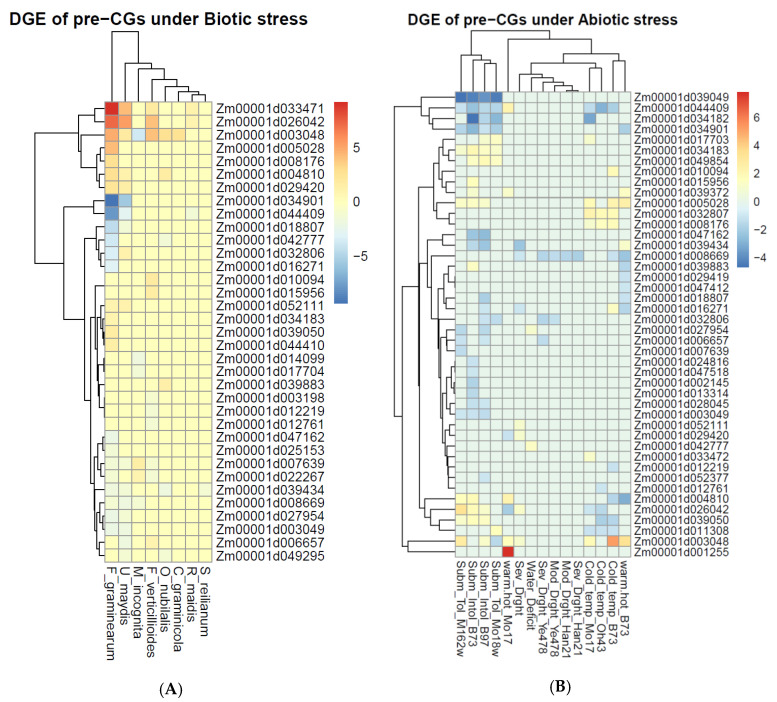
Differential gene expression (DGE) of pre-candidate genes (CGs) under (**A**) different biotic agents including *Fusarium graminearum* and *verticelloides*, *Meloidogyne incognita*, *Ostrinia nubilalis*, *Rhopalosiphum maidis*, and *Ustilago maydis*, and (**B**) abiotic stress conditions such as cold temperature, drought, heat, and submergence.

The in silico analyses revealed that 62 pre-CGs were differentially expressed under biotic and abiotic stress conditions, suggesting a probable involvement in plant defense mechanisms. The in silico analyses conducted using the GENEVESTIGOR software showed that 58 of the 68 pre-CGs that had expression data were differentially expressed at different plant developmental stages (Figure S5). These were at the seedling, inflorescence formation, and ear formation developmental stages under biotic stress conditions (Figure S5A) and at the germination, seedling, stem elongation, and anthesis developmental stages under abiotic stress conditions (Figure S5B). The expression of the pre-CGs was also modified in organs relevant to FAW feeding and at the ear development stage (Figure S6) under both biotic (Figure S6A) and abiotic stress conditions (Figure S6B). The 58 pre-CGs were also differentially expressed in the “Perturbation” analyses when subjected to biotic stressors like *Colletotrichum graminicola*, *Cercospora zeina*, *Fusarium verticilloides*, *Rhopalosiphum maidis*, and also jasmonates and jasmonate-like chemicals (Figure S7A), and abiotic stresses such as cold, dehydration, drought, heat, and submergence (Figure S7B). The gene expression atlas (GXA) platform revealed 52 pre-CGs differentially expressed under stress conditions (Figure 5). Thirty-five pre-CGs were induced by biotic stress (Figure 5A), 44 by abiotic stress (Figure 5B), and of these two groups, 27 by both conditions. Most of the pre-CGs were upregulated under biotic and downregulated under abiotic stress conditions. The biotic stressors utilized in the GXA include those in the GENEVESTIGATOR (except *Cercospora zeina*) in addition to *Fusarium graminnearum*, *Meloidogyne incognita*, *Sporisorium reilianum*, *Ustilago maydis*, and the stem borer *Ostrinia nubilalis* (the European corn borer, ECB), and the two platforms shared similar abiotic stress conditions. 

### 5.6. Pre-CGs were Co-Expressed with Their Rice and Arabidopsis Thaliana Ortholog Genes

**Figure 6:**
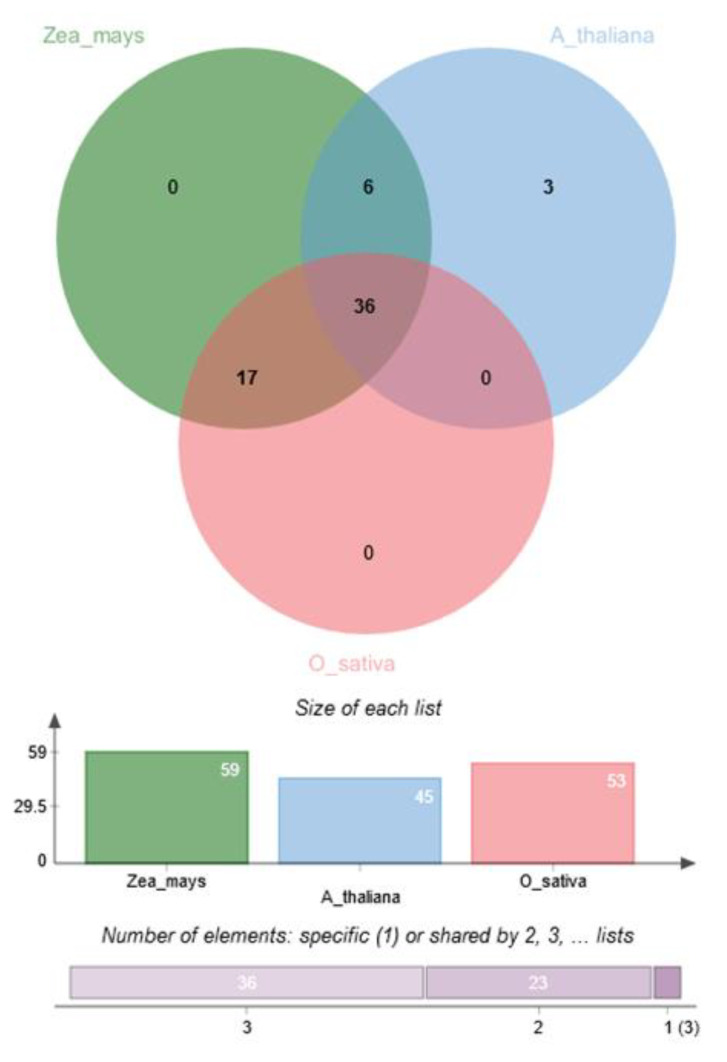
Venn diagram showing the co-expression clusters and overlaps between maize (Zea_mays) and their rice (O_sativa) and *Arabidopsis* (A_thaliana) orthologs. Co-expression between maize pre-CG and rice and *Arabidopsis thaliana* orthologs.

**Figure 7:**
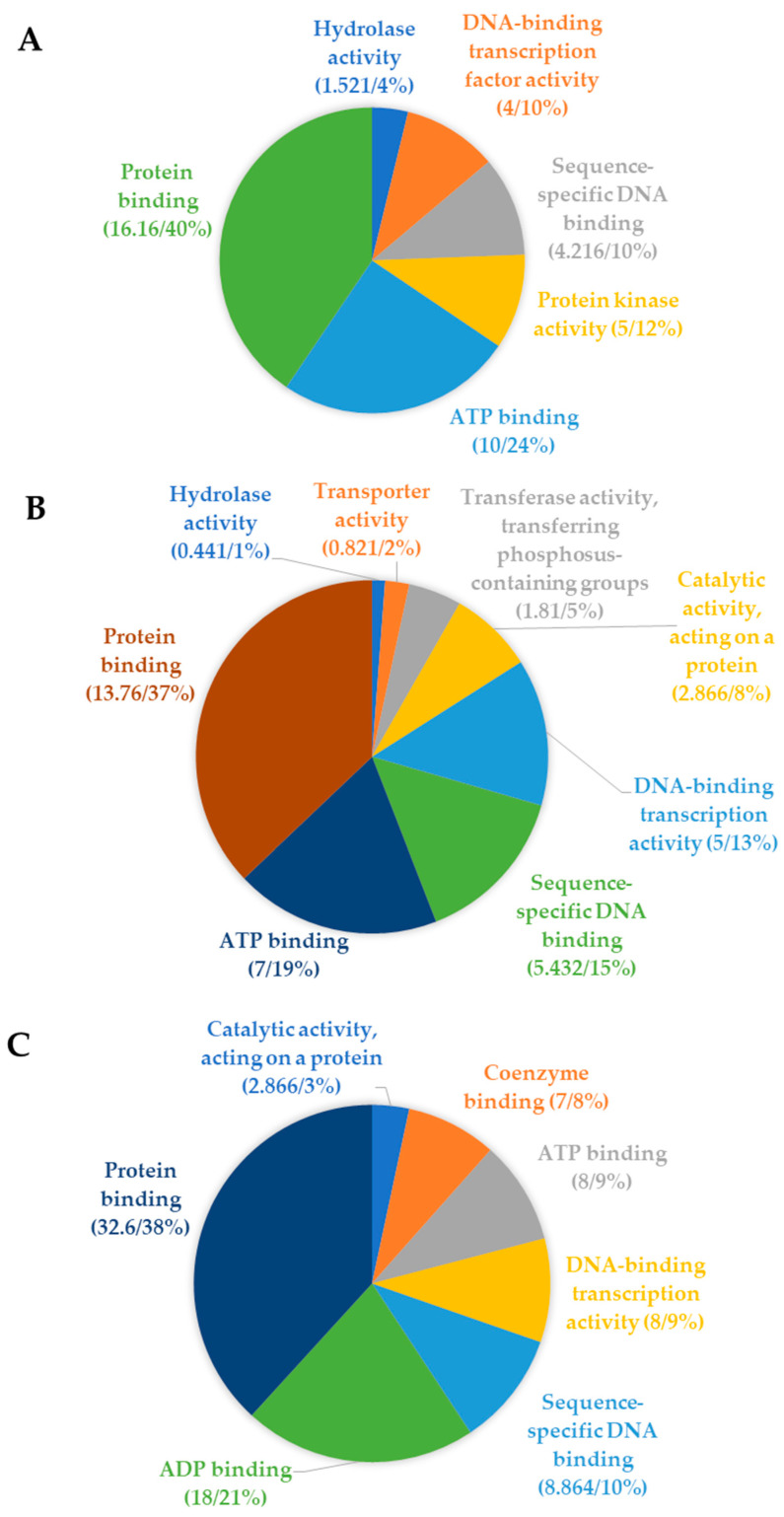
Distribution of the molecular functions enriched within the pre-CG (**A**) and their *A. thaliana* (**B**) and rice (**C**) orthologs as revealed by the gene ontology mapping.

The co-expression analysis between the pre-CGs and their rice and *Arabidopsis thaliana orthologs* showed that all maize genes co-expressed with at least one ortholog from either or both rice and *Arabidopsis* (Figure 6). Thirty-six co-expression clusters were common to all three species while 17 and 6 groups were common to maize and rice, and maize and *Arabidopsis*, respectively. Three clusters comprising 10 genes were unique to *Arabidopsis* alone and there was no cluster shared uniquely between rice and *Arabidopsis* (Figure 6). The functional gene ontology (GO) categories enriched by the maize pre-GGs (Figure 7A) and their rice (Figure 7B) and *Arabidopsis* (Figure 7C) orthologs were similar and pertained mostly to protein kinase and DNA-binding molecular functions. Based on the co-expression and GO functional term similarities between maize pre-CGs and their rice and Arabidopsis thaliana orthologs, 62 pre-CGs were classified as possibly functionally involved in maize plant defense mechanisms (Table S2).

### 5.7. NbCGs were Biologically Connected to the GbCGs

**Figure 8:**
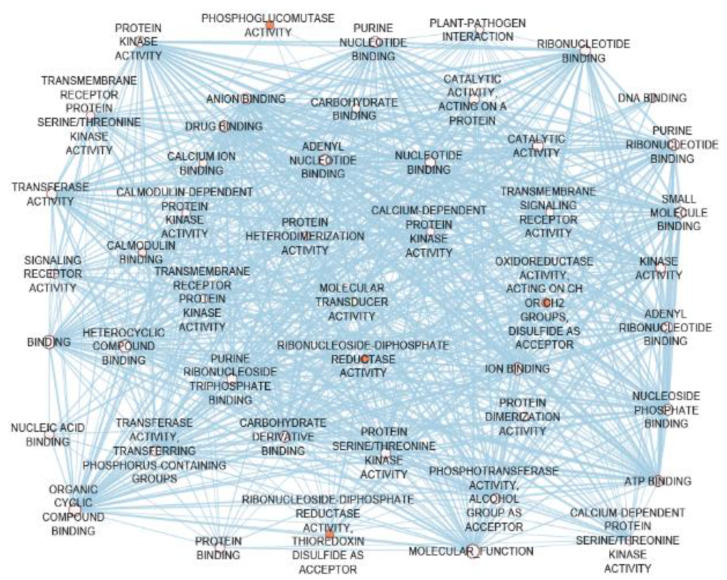
Molecular function network showing interactions (edges) among the gene ontology terms (nodes) enriched within the candidate genes.

Based on the CG prioritization criteria, 64 pre-CGs showed at least one evidence of involvement in plant defense mechanisms, of which, 55 had two or more evidence (Table S2), and therefore were considered as GbCGs. These GbCGs (guide genes) were used to discover NbCGs within the MIRGRs. In total, 3737 NbCGs biologically connected to the GbCGs were discovered, of which, 730 had a connectivity score of more than 5. Of the 730 NbCGs, 242 were differentially expressed under biotic and abiotic stress conditions (Figure S8) and most were upregulated when exposed to biotic agents (Figure S8A) and downregulated when the plant faced abiotic stressors (Figure S8B). Further, 107 of these differentially expressed NbCGs are located within the MIRGRs (Table S3). More than half of these 107 NbCGs were enriched with biological process GO terms relevant to plant defense mechanisms. The biological connections that exist among the two groups of CGs were further illustrated by the GO terms for the molecular functions enriched within these CGs (Figure 8). The functions displayed by the CGs include plant defense-associated GO terms such as protein kinase activities, DNA, ATP, ion, protein binding factors, oxydoreduction activities, signaling transduction factors, and calcium-dependent channels (Figure 8). These interactions were further vindicated at the proteomic level by the existence of protein–protein interactions among the CGs (Figure S9), suggesting their involvement in the network-like defense mechanism against insect damages.

## 6. Discussion and Conclusions

### 6.1. Association Mapping Panel

In this study, a diverse association mapping panel (AMP) composed of maize lines adapted to African environments was evaluated in three environments (in Kasese in season 2017B, and in Namulonge in seasons 2018A and 2019A) for FAW damage resistance and the bulked grains from each genotype were subjected to an MW bioassay. The lines that composed the AMP were bred in Uganda, Kenya, and Nigeria, and displayed a genetic and geographical diversity suitable not only for association mapping but also would be of great use in ongoing maize breeding projects. The majority of these lines were developed for resistance to either stem borers or storage pests by CIMMYT of Nairobi [39,40,41] and IITA of Ibadan, or, in the case of DH lines from CIMMYT, from crosses involving either a stem borer or a storage pest-resistant line. All the resistance traits recorded were highly significantly varied among the lines of the AMP, owing to the high genetic diversity present in the AMP. The observed genetic variability was of paramount interest, especially for FAW since the lines in the AMP were not originally developed for resistance to this insect pest. The observed genetic variability for FAW resistance could be a consequence of the genetic correlations between maize resistance mechanisms to FAW and stem borers [4,68,69,82,83] but also could have been retained by the lines of the AMP during their development since this trait was not a selection target. The moderate to high estimates of heritability and the high genetic variability obtained in this study shows the suitability of the measured traits for improving both MW and FAW resistance in maize and their potential for association mapping studies. The AMP could serve as a base population for multiple insect resistance breeding targeting FAW, stem borers, and storage pests which are hazardous threats to food security in sub-Saharan Africa [1,5,84,85]. Since the environmental effect and the interactions between the environment and the genotypes were significant for FAW damage resistance, the AMP needs to be evaluated in wider multi-environment trials to assess the stability status of the lines in the panel for these target traits across national and regional locations, seasons, and years, so as to aid in making the best breeding decisions [86,87]. Secondary metabolites such as cell wall constituents and proteins are essential for resistance to storage pests and their accumulation in the grain is affected by environmental parameters [12,88,89,90]. Therefore, it is necessary to evaluate the AMP for MW resistance traits in several individual environments and increase the sample size so as to perform both single and across-environments analyses to better inform future resistance breeding programs.

### 6.2. Linkage Disequilibrium and Control of False-Positive and Negative Association

The LD decayed rapidly in the AMP at distances of 22.7 and 7.92 at a cut-off *r*^2^ of 0.1 and 0.2, respectively, indicating a high recombination rate and promising high resolution in GWAS [91], which is in line with the faster LD decay characterizing tropical maize lines [91,92]. Chaikam et al. [93] found on a maize panel composed of lines adapted to tropical and subtropical ecologies that the average LD decayed at 27.31 and 9.48 kb at *r*^2^ = 0.1 and 0.2, respectively, which is very similar to the results presented in the current study.

The high genetic and geographic diversity in the AMP resulted in a pronounced population structure that was necessary to account for in the GWAS analyses for FAW and MW resistance traits to avoid false positive and negative association signals [30]. Two strategies were used to limit the chances of getting false positive and negative associations and to increase the statistical power of the QTN discovery. To reduce the multiple-testing burden, an LD-based pruning approach was used [94]. Since the population structure may affect traits in a population differently, accounting for it is not straightforward [30,64], and thus it was not realistic to include a fixed number of PCs to analyze different traits with varying phenotypic correlations. Therefore, a different number of PCs was fitted in the GWAS model for each trait, and the Manhattan and Q–Q plots were investigated to evaluate the level of control of false positives and negatives [30]. As a result, several high-confidence SNP–trait associations at a BGSL of 5% (47 QTNs) and at the highest level of 1% (31 QTNs) were discovered, proving the worthiness of these quality control approaches used.

### 6.3. QTNs for Both Single and Combined Maize Resistance to FAW and MW

This study is the first reported GWAS for maize resistance to FAW and MW as all previous reports used bi-parental QTL mapping studies [18,67,68,69,95,96]. Sixty-two QTNs significantly (BGSL > 30%) associated with maize resistance to MW and FAW were discovered across all the 10 maize chromosomes. However, no QTN for maize resistance to FAW damage was discovered on chromosomes 6 and 7. Fourteen QTNs were associated with resistance to FAW, of which, nine were discovered at a BGSL of 5%. Bi-parental QTL analyses conducted previously for FAW resistance identified less QTL than reported in this study [67,68,69]. Seven QTL were discovered by Brooks et al. [68,69] including on chromosomes 6 and 7 from populations derived from crosses Mp708 × Mo17 and A619 × Mp708, respectively. Womack et al. [67] identified six QTL including one on chromosome 7 on the same population as studied by Brooks et al. [68]. Several FAW resistance QTL discovered in these three studies (three in Brooks et al. [68], one in Brooks et al. [69], and four in Womack et al. [67]) co-localize with 6 of the 14 QTNs for resistance to FAW identified in this study in maize bins 1.09, 2.02, 5.04, 8.03, and 10.04, and some of these regions were also reported to be associated with maize resistance to the southwestern corn borer [67,68,69].

### 6.4. Resistance Across Insect Pest Species and Maize Organs

The majority of FAW and MW resistance QTNs fell within or very close to MIRGRs, corroborating the previous meta-analysis results for the commonality of resistance regions across maize organs, namely leaves, stems, and kernels, and across insect pest species [16]. This is further vindicated in this study with the discovery of six QTNs associated with resistance to both FAW leaf damage and MW grain damage, of which, four are located within the MIRGRs. The nature of the genetic action of these multiple insect resistance-associated QTNs could either be based on gene pleiotropy or close linkage [97]. Furthermore, a probable role of maize biochemical components such as benzoxazinoids and cell wall constituents is further illustrated with the colocalization of related QTLs with the MIRGRs previously presented by Badji et al. [16]. These maize biochemicals were found to play essential roles in maize resistance to a range of insect species including stem borers, FAW, and MW [98,99,100]. Regardless of whether gene pleiotropy or close linkage, these MIRGRs, once validated in diverse backgrounds, could be used in GAB to develop combined resistance in maize varieties adapted to local environments and consumer needs. These comparative mapping results are further supported by the outcome of the CG identification and prioritization analyses.

### 6.5. Promising CGs for Maize Resistance to Multiple-Insect Pests

The identification and prioritization of CGs is an essential post-GWAS analysis to identify genes in the vicinity of QTNs that have the highest likelihood of association with traits of interest. In species like maize that present extensive genomic information stored in various databases and that share common evolutionary signatures with closely or distantly related species with equal or even more comprehensive functional characterization, integrative approaches hold tremendous promise for the discovery and validation of meaningful causal genes for several traits of economic importance [33,34,101]. In that vein, the current study was also intended to discover and prioritize CGs associated with traits for maize resistance to insect pests. In total, 78 pre-CGs were discovered around the QTNs, of which, 62 were given priority based on their functional information.

Five of the six QTNs identified for combined FAW and MW resistance were associated with one gene each, further suggesting a possible pleiotropic genetic implication in the regulation of multiple insect resistance, and therefore presenting great interest for multiple insect resistance breeding. Pleiotropy, where one gene regulates the expression of more than one phenotype, is pervasive in the control of complex traits such as resistance to insect pests even when traits are not positively correlated [102,103,104,105]. Four QTNs, 4593663-22-G/A on chromosome 3 for GWL and FP, 4587005-7-C/G for AK-NH and 4579331-18-T/C for AP on chromosome 6, and 4776702-53-G/A on chromosome 10 for AK, did not have any pre-CG within the 30 kb window. Possibly, these QTNs resulted from spurious associations that were not successfully controlled during the GWAS analysis.

Furthermore, network-based inferences are pivotal in studies aimed at finding functional pathways regulating genes and are instrumental in discovering additional genes connected to predefined genes associated with traits of interest through diverse analyses such as association mapping experiments [28,106,107]. Therefore, through a network-based inference approach, an additional 107 genes, subsetted from a total of 3737 genes biologically connected to the GbCGs, were differentially expressed under either biotic or abiotic stress conditions or both and located within MIRGRs previously reported [16].

The genomic and functional information related to most of these CGs, the conserved domains within their protein sequences, and genetic descriptions of their co-expressed rice and arabidopsis orthologs suggest their possible involvement in plant defense mechanisms. Functional features known for their involvement in both biotic and abiotic plant response such as WRKY, F-BOX, NAM, bZIP, LRR, AUX_IAA, zf-C2H2, DNA, ATP, ion, protein-binding protein domains, MADS-box, C2C2-YABBY, MYB transcription factors, calcium-related transmembrane transport elements, protein kinases, oxydoreduction activities, and several binding factors [108,109,110,111,112,113] characterized most of the CGs, making them promising genetic factors for the regulation of plant response to insect pests.

Most of the CGs had modified expressions under several biotic stress conditions including infection with the European corn borer (*Ostrinia nubilalis*), and on maize organs and at developmental stages relevant to FAW feeding. The expression of GWAS-based CGs in maize ear-related organs and developmental stages indicated that these genes could have an influence on the accumulation of assimilates in the grain, among which were phenolic compounds critical for resistance to storage pests such as MW [89,114,115]. In agreement with the negative interaction between plant biotic and abiotic stress responses resulting from negative cross-talk between absicic acid (ABA) and the jasmonic acid (JA), salicylic acid (SA), and ethylene (ET) signaling pathways [108,109,110,112,116,117], most of the CGs were upregulated and downregulated under biotic and abiotic stress conditions, respectively. In vivo expression analyses under local conditions would help to confirm the role of these CGs in maize response to FAW, SB, and MW.

Evidence of involvement of the CGs in network-like defense mechanisms were provided by the existence of protein–protein interactions among them. These interactions were expected since plant defense mechanisms against insect herbivores are complex mechanisms that integrate signaling molecules, hormones, and transcription factors, that collaborate as a network under the regulation of signaling molecules such as ABA, JA, SA, and ET, among others, to modulate the production of secondary metabolites for direct and indirect responses to insect damage [108,109,110,116,118,119,120,121]. The GO molecular function network which was constituted of 47 GO terms, interconnected by 759 edges enriched by these CGs, further illustrated the extent of interaction among these genes and supports their involvement in network-like defense mechanisms against biotic and abiotic stresses. These GO terms were related to protein kinase activities, binding factors, oxidoreduction activities, and plant–pathogen interactions, further indicating that they may play crucial roles in maize resistance to insect pests such as FAW, MW, and SB.

### 6.6. Research and Breeding Perspectives

Considering the high economic importance of multiple insect pest species including stem borers, FAW, and MW in terms of fodder and grain yield loss and mycotoxin contamination [5,6], the genetic resistance information revealed in this study would be of great use in genomics-aided breeding activities targeting the selection of promising lines and the development of varieties with good levels of resistance to either single or multiple insect species.

The QTN/QTL information along with the putative CGs discovered in this study are worth going through further validation steps in more diverse genetic and environmental backgrounds and through in vivo analyses involving a differential gene expression and gene knock-out or silencing techniques, or fine-mapping activities and gene editing, among others [122]. Further, once validated under local conditions, this resistance-related genetic information would further improve the capabilities of molecular breeding and genetic engineering programs targeted at building insect resistance in maize lines of agronomic importance in Africa.

The plethora of genomic regions and genes putatively involved in resistance corroborates the complex architecture of resistance. The co-localization of genomic regions associated with resistance to several insect pest species in different maize organs and biological and functional connections among prioritized candidate genes under these regions indicates that multiple insect resistance could be governed by pleiotropy. This pleiotropic effect could be characterized by complex networks involving pathways responsible for the production of defensive biochemicals such as benzoxazinoids and cell wall constituents [89,99,100,108]. Studies allowing direct investigations of the role of these biochemicals in maize resistance to multiple insect pests, especially in reference to their possible pleiotropy, should be carried out along with validation steps needed for the resistance genetic information presented in the current research.

Furthermore, the polygenic nature of the resistance traits studied here indicates that MAS alone might not be efficient for resistance breeding [123,124]. The efficiency of genomic selection, a complementary approach to GWAS and MAS that uses whole genome markers to achieve selection on a collection of unphenotyped germplasm [125], is worth investigating in the AMP, owing to the fairly high LD within the genetic data and moderate to high heritabilities of the resistance traits investigated in the current study [126,127].

## Figures and Tables

**Table 1:** Analysis of variance for maize resistance to fall armyworm (FAW) damage in Kasese 2017B (2017B), in Namulonge 2018A (2018A), and 2019A (2019A) and across environments (Across Env.).

SOURCE OF VARIATION	DF	2017B	DF	2019A	DF	2018A	DF	ACROSS ENV.
GENOTYPE	315	1.51 **	251	3.12 ***	91	3.48 ***	357	2.76 ***
BLOCK	11	3.75 ***	9	1.59 ns	4	7.17 ***	11	2.41 *
REPLICATION					1	26.95 ***		
ENVIRONMENT							2	270.57 ***
GENOTYPE*ENVIRONMENT							300	2.14 ***
RESIDUALS	9	0.25	49	0.90	123	1.12	195	1.12
H^2^		0.80		0.72		0.67		0.25

Significance codes: 0.001 ‘***’ 0.01 ‘**’ 0.5 ‘*’. Df = degrees of freedom; H^2^ = entry mean-based broad-sense heritability, ns = non significant. 2017B = Kasese 2017B; 2018A = Namulonge 2018A; 2019A = Namulonge 2019A; and Across Env. = across environments.

**Table 2:** Results of the analysis of variance for maize weevil (MW) resistance traits.

SOURCE OF VARIATION	DF	AK	AP	FP	GWL	NH
GENOTYPE	131	4795.47 ***	5947.91 ***	2.56 ***	19.11 ***	13070.89 ***
REPLICATION	2	3668.67 ns	1215.68 *	0.07 ns	1.54 ns	2660.36 *
RESIDUALS	200	1218.15	1383.90	0.16	2.83	3417.45
H^2^		0.79	0.79	0.95	0.87	0.78

Significance codes: 0.000 ‘***’ 0.001 ‘**’ 0.1 ‘*’. AK = number of affected kernels, NH = number grain holes, AP = number of emerged adult progenies, FP = total amount of flour produced, and GWL = grain weight loss. Df = degrees of freedom; H^2^ = entry mean-based broad sense heritability.

**Table 3:** List of the 62 quantitative trait nucleotides (QTNs) associated with resistance to fall armyworm (FAW) damage and maize weevil (MW) traits.

Chr.Bin	Position	SNP-Alleles ^a^	*p-*Value	Effect	Trait	BGSL
**1.02**	18,282,139	2544389-10-G/C	8.49 × 10^−5^	−0.61926	GWL	0.3
**1.02**	21,511,322	2399751-6-C/A	3.95 × 10^−6^	0.397572	AP	0.05
**1.04**	69,429,238	5584129-55-C/T	1.20 × 10^−13^	−1.34659	AK	0.01
**1.04**	69,747,754	4580363-8-A/G	1.79 × 10^−5^	−0.45677	AK	0.3
**1.08**	238,892,103	4583673-29-G/C	6.29 × 10^−6^	−0.45143	GWL	0.05
**1.09**	**263,624,976**	**100024832-19-A/C**	**3.83 × 10^−7^**	**−1.39669**	**GWL**	**0.01**
			**6.36 × 10^−5^**	**−0.56467**	**FAW**	**0.3**
**1.09**	264,933,475	4583685-9-G/A	4.88 × 10^−5^	−0.56303	NH	0.3
			5.98 × 10^−5^	−0.59983	AK	0.3
**1.11**	285,936,150	4580090-67-T/C	8.37 × 10^−7^	0.796615	GWL	0.01
**1.12**	305,156,544	2382596-67-A/G	1.63 × 10^−10^	−0.36002	FAW	0.01
**2.02**	6,741,658	2452223-17-A/G	6.79 × 10^−6^	0.213179	FAW	0.05
**2.04**	30,341,425	4771831-60-G/T	2.22 × 10^−6^	−0.61067	AK	0.01
**2.04**	35,377,279	4767220-53-G/A	1.64 × 10^−5^	−0.52503	AK	0.3
**2.04**	40,608,209	2388222-45-G/C	1.01 × 10^-9^	0.473004	FP	0.01
			1.35 × 10^-9^	0.752083	GWL	0.01
**2.05**	140,747,202	2435073-40-T/C	3.13 × 10^-6^	−0.41627	AP	0.01
			7.09 × 10^-5^	0.289661	FP	0.3
**2.06**	154,630,564	2448649-48-G/A	5.41 × 10^-5^	−0.18627	FAW	0.3
**2.08**	213,714,960	4583437-30-G/C	3.16 × 10^-5^	0.195599	FP	0.3
**2.08**	221,951,608	4765698-16-A/G	2.42 × 10^−5^	−1.02779	AK	0.3
**2.10**	236,778,497	100130818-44-A/G	1.70 × 10^−5^	0.391072	FP	0.3
**2.10**	236,789,029	4591349-29-A/G	5.73 × 10^−6^	0.366018	GWL	0.05
**3.01**	**2,734,515**	**9714175-54-C/G**	**1.74 × 10^−8^**	**−0.52816**	**FAW**	**0.01**
			**1.88 × 10^−5^**	**0.885829**	**NH**	**0.3**
**3.02**	**4,141,348**	**4764930-10-C/T**	**3.43 × 10^−7^**	**−0.51944**	**FAW**	**0.01**
			**1.14 × 10^−5^**	**−1.03199**	**GWL**	**0.05**
**3.04**	17,591,392	4772102-17-T/G	2.15 × 10^−5^	0.305217	FP	0.3
**3.04**	71,004,409	4593663-22-G/A	2.15 × 10^−10^	0.966386	GWL	0.01
			2.55 × 10^−7^	0.48656	FP	0.01
**3.06**	179,391,224	2446859-65-C/G	9.98 × 10^−6^	−0.30607	AP	0.05
**3.07**	201,766,146	4584446-12-G/C	1.08 × 10^−6^	0.493102	NH	0.01
**3.09**	227,436,274	4583173-13-T/C	9.21 × 10^−6^	−0.62512	GWL	0.05
**4.03**	19,181,255	2381322-13-C/G	5.34 × 10^−9^	0.202627	FAW	0.01
**4.04**	24,984,097	4779016-24-C/T	5.56 × 10^−5^	0.468163	NH	0.3
**4.05**	48,323,977	4577027-47-G/A	1.75 × 10^−5^	0.551228	GWL	0.3
**4.05**	**78,882,987**	**100220678-45-A/G**	**9.85 × 10^−6^**	**0.184883**	**FAW**	**0.05**
			**7.12 × 10^−5^**	**0.324198**	**FP**	**0.3**
**4.08**	180,072,262	4771330-29-T/C	3.40 × 10^−7^	−0.8024	NH	0.01
**4.08**	188,548,237	2619648-16-T/C	3.79 × 10^−6^	−0.91698	GWL	0.05
**5.02**	8,372,190	4589321-22-G/A	7.07 × 10^−6^	−0.67142	AK	0.05
**5.03**	32,460,125	7048960-37-T/G	1.22 × 10^−6^	−0.75462	NH	0.01
**5.04**	134,168,179	7049219-26-T/C	5.10 × 10^−5^	0.16596	FAW	0.3
**5.04**	155,012,378	4584182-35-C/G	2.64 × 10^−5^	−0.16674	FAW	0.3
**5.07**	204,689,646	4774140-50-G/A	1.51 × 10^−5^	0.372348	FP	0.05
**6.01**	9,188,598	4587005-7-C/G	2.68 × 10^−6^	−0.65839	AK	0.01
			8.38 × 10^−5^	−0.41379	NH	0.3
**6.01**	77,513,355	4771590-67-A/T	4.69 × 10^−5^	0.299541	FP	0.3
**6.03**	103,106,812	5586936-13-T/C	5.07 × 10^−5^	0.312373	FP	0.3
**6.06**	157,597,555	4579331-18-T/C	1.90 × 10^−6^	0.544151	AP	0.01
**6.08**	169,246,523	4764931-6-G/A	5.19 × 10^−6^	−0.61458	FP	0.05
			8.92 × 10^−5^	−0.66184	AP	0.3
**7.01**	5,750,453	4771072-39-A/G	6.80 × 10^−5^	0.428352	GWL	0.3
**7.03**	152,580,067	5587204-51-A/C	2.45 × 10^−5^	−1.30905	AK	0.3
**7.05**	173,989,867	4580355-27-G/A	4.84 × 10^−7^	−0.57199	GWL	0.01
			5.08 × 10^−7^	0.406797	AP	0.01
**8.00**	328,928	4773640-63-T/A	7.16 × 10^−8^	0.34059	FP	0.01
**8.02**	16,558,612	4770550-8-G/C	6.47 × 10^−6^	0.374068	GWL	0.05
**8.03**	99,111,439	2504966-32-A/G	9.62 × 10^−6^	0.264805	FAW	0.05
**8.05**	**146,321,767**	**2559495-18-T/G**	**6.26 × 10^−5^**	**−0.15544**	**FAW**	**0.3**
			**8.31 × 10^−5^**	**−0.54446**	**AK**	**0.3**
**8.08**	170,354,517	2610943-54-T/C	3.08 × 10^−6^	0.570008	GWL	0.01
			9.53 × 10^−5^	−0.37079	AP	0.3
**8.09**	176,518,972	2376195-62-T/G	7.58 × 10^−5^	0.393732	FP	0.3
**8.09**	180,177,242	4579847-66-T/G	6.92 × 10^−5^	0.277417	FP	0.3
**9.03**	61,164,617	4771587-19-T/C	6.24 × 10^−7^	−1.9427	AK	0.01
**9.04**	120,457,334	100023814-29-T/G	4.20 × 10^−5^	−0.52317	AK	0.3
			8.2 × 10^−5^	0.288404	FP	0.3
**9.05**	**129,393,054**	**9682691-38-C/T**	**5.68 × 10^−10^**	**0.797225**	**FP**	**0.01**
			**4.41 × 10^−6^**	**−0.34413**	**FAW**	**0.05**
**9.05**	133,252,665	4764675-42-C/G	2.69 × 10^−5^	−0.52765	AP	0.3
**9.06**	141,396,844	2425091-21-G/A	4.50 × 10^−10^	0.689575	NH	0.01
**10.04**	89,412,526	4582917-12-A/G	1.05 × 10^−7^	0.571392	GWL	0.01
**10.04**	99,693,244	2539012-9-A/C	2.39 × 10^−10^	0.767475	GWL	0.01
			1.87 × 10^−6^	0.363841	FP	0.01
			3.21 × 10^−6^	−0.44639	AP	0.05
**10.04**	106,804,143	100298755-56-T/C	2.20 × 10^−8^	−0.31497	FAW	0.01
**10.04**	125,628,521	4776702-53-G/A	1.89 × 10^−7^	1.750698	AK	0.01
**10.05**	136,798,456	7061499-37-A/G	1.29 × 10^−12^	0.547738	AP	0.01

^a^ The allele before the slash (/) increases the trait and the allele after the slash decreases the trait. Bonferroni genome-wide significance level (BGSL). AK = number of affected kernels, NH = number grain holes, AP = number of emerged adult progenies, FP = total amount of flour produced, and GWL = grain weight loss. QTNs in bold are associated with both FAW and MW resistance.

## Supplementary Materials

The following are available online at https://www.mdpi.com/2073-4425/11/6/689/s1. Table S1. Descriptions of parents and crosses that constituted the doubled-haploid population. Table S2. Candidate genes located in the vicinity of the quantitative trait nucleotides (QTNs) along with their genetic information. Table S3. 107 Network-CGs with their chromosome (Chr), start and end position based on the AGPv4 maize genome reference genome and descriptions. Figure S1. Distribution of the 3124 LDPSNPs across the 10 maize chromosomes. Figure S2. Rating of maize plants based on foliar damage by FAW. Figure S3. Number of principal components (PC) included in the GWAS model for fall armyworm (FAW) damage, and for the different maize weevil (MW) resistance traits: number of affected kernels (AK), number grain holes (NH), number of emerger adult progenies (AP), total amount of flour produced (FP), and grain weight loss (GWL). Figure S4. Physical map based on the AGPv4 maize reference genome showing on the chromosomes the single (in green) and multiple (in blue) insect resistance genomic regions (IRGR) and on the side their colocalizations with QTL for Asian corn borer (ACB), southwestern corn borer (SWCB), Fall armyworm (FAW), Maize weevil (MW), African maize stalk borer (AMSB), spotted SB (SSB) resistance, and maize benzoxazinoids (Benzox) content. Figure S5. *In-silico* expression profile of the CG at different maize developmental stages relevant to FAW and MW damage under different biotic (A) and abiotic (B) stress conditions. Figure S6. *In-silico* expression profile of the CG in different maize organs relevant to FAW and MW damage under different biotic (A) and abiotic (B) stress conditions. Figure S7. *In-silico* expression profile of the CG under different biotic stress, jamonates and jasmonate-like treatments (A) and abiotic (B) stress conditions. Figure S8. Network-based candidate genes differentially expressed under different biotic agents (A) including *Fusarium graminearum* and *verticelloides, Meloidogyne incognita, Ostrinia nubilalis, Rhopalosiphum maidis* and *Ustilago maydis*, and under different abiotic stress conditions (B) such as cold temperature, drought, heat, and submergence. Figure S9. Protein-protein interaction network (edges) linking the CGs (nodes).
