# Supplementary material for: Genetic Basis of Maize Resistance to Multiple Insect Pests: Integrated Genome-Wide Comparative Mapping and Candidate Gene Prioritization
Source: Genes (Basel). 2020 Jun 24;11(6):689. doi: 10.3390/genes11060689 (PMC7349181; doi:10.3390/genes11060689)
Supplement: Supplementary file 1 [file genes-11-00689-s001.pdf]

SUPPLEMENTARY: TABLES AND FIGURES

Table S1. Descriptions of parents and crosses that constituted the doubled-haploid population

Parents of the doubled-haploid population					
	**Descriptions**	**CKSBL10007**	**CKSBL10011**	**CKSBL10203**	**CKSPL10066**
**CKSBL10007**	CIMMYT Stem borer-resistant line				
**CKSBL10011**	CIMMYT Stem borer-resistant line				
**CKSBL10203**	CIMMYT Stem borer-resistant line				
**CKSPL10066**	CIMMYT Storage pest- resistant line		X		
**CML312**	CIMMYT elite line	X		X	
**CML485**	CIMMYT elite line				X

X=Crosses between lines

Table S2. Candidate genes located in the vicinity of the quantitative trait nucleotides (QTNs) along with their genetic information.

**Chr.**	**QTNs**	**Trait**	**Candidate Gene**	**DQTN**	**CGC**	**Gene description**	**Conserved protein domain**
**1**	2544389-10-G/C	GWL	Zm00001d027955	IG	A	MADS-box transcription factor 47	K-box superfamily
			Zm00001d027954	-1,862	B	Expressed protein	TMEM131_like || Herpes_BLLF1 superfamily
	2399751-6-C/A	AP	Zm00001d028046	2,523	A	Putative pentatricopeptide repeat-containing protein	PLN3218 superfamily
			Zm00001d028045	IG	A	Mannose-1-phosphate guanylyltransferase 1	M1P_guanylylT_A_like_N || LbetaH superfamily
	5584129-55-C/T	AK	Zm00001d029411	-1,217	NGI	NA	NA
			Zm00001d029412	IG	A	Patatin-like protein 2	Pat17_isozyme_like
	4580363-8-A/G	AK	Zm00001d029419	IG	A	Probable protein phosphatase 2C 12	PP2Cc
			Zm00001d029420	2,023	A	Protein WEAK CHLOROPLAST MOVEMENT UNDER BLUE LIGHT 1	WEMBL
	4583673-29-G/C	GWL	Zm00001d032806	589	A	Pectate lyase 12	Amb_all

Chr.	QTNs	Trait	Candidate Gene	DQTN	CGC	Gene description	Conserved protein domain
			Zm00001d032807	4,404	A	RNA-binding (RRM/RBD/RNP motifs) family protein	RRM1_SF3B4
	**100024832-19-A/C**	**GWL-FAW**	**Zm00001d033471**	**-3,094**	**A**	**Putative DNA-binding protein**	WRKY
			**Zm00001d033472**	**IG**	**A**	**TATA-binding protein1**	PLN62
	4583685-9-G/A	NH-AK	Zm00001d033508	IG	A	C2C2-YABBY-transcription factor 12	YABBY
	4580090-67-T/C	GWL	Zm00001d034182	-4,182	B	NA	NA
			Zm00001d034183	IG	A	clast3-related	PAC2
	2382596-67-A/G	FAW	Zm00001d034901	-219	A	Receptor protein kinase-like protein	SASA
**2**	2452223-17-A/G	FAW	Zm00001d002145	IG	B	Formation of crista junctions protein 1	Mitofilin superfamily
	4771831-60-G/T	AK	Zm00001d003048	IG	A	Disease resistance gene analog PIC17	NB-ARC superfamily || LRR_8|| LRR
			Zm00001d003049	3,867	A	Casein kinase 1-like protein 6	STKc_CK1_delta_epsilon
	4767220-53-G/A	AK	Zm00001d003198	IG	A	eIF-2-alpha kinase GCN2	STKc_EIF2AK4_GCN2_rpt2 || PLN2972 superfamily || RWD
	2388222-45-G/C	FP-GWL	Zm00001d003335	-767	C	OSJNBaNANA89K21.8 protein	zf-C2H2_6
	2435073-40-T/C	AP-FP	Zm00001d004810	IG	A	FHA domain-containing protein PS1	PIN_Smg5-6-like || FHA
	2448649-48-G/A	FAW	Zm00001d005028	IG	A	NAC domain-containing protein 77	NAM
	4583437-30-G/C	FP	Zm00001d006657	IG	A	Homogentisate solanesyltransferase chloroplastic	PT_UbiA_HPT1
	4765698-16-A/G	AK	Zm00001d007088	-3,974	C	Retrovirus-related Pol polyprotein LINE-1	NA
			Zm00001d007087	-4,180	C	NA	NA
			Zm00001d007089	IG	A	Protein CHROMATIN REMODELING 5	PLN3142 superfamily || CD1_tandem || DUF428 || CD_CSD superfamily
	100130818-44-A/G	FP	Zm00001d007639	IG	A	GTP-binding family protein	PRK93 || GTP_HflX superfamily
	4591349-29-A/G	GWL	Zm00001d007640	-2,040	A	F-box/LRR-repeat protein 4	AMN1 superfamily

Chr.	QTNs	Trait	Candidate Gene	DQTN	CGC	Gene description	Conserved protein domain
**3**	**9714175-54-C/G**	**FAW-NH**	**Zm00001d039372**	**3,380**	**A**	**Small nuclear ribonucleoprotein family protein**	Sm_E
	**4764930-10-C/T**	**FAW-GWL**	**Zm00001d039434**	**IG**	**A**	**Agamous-like MADS-box protein AGL5**	MADS_MEF2_like || K-box
	4772102-17-T/G	FP	Zm00001d039883	IG	A	Mitochondrial glycoprotein family protein	MAM33
	4593663-22-G/A	GWL-FP	NA	NA	NCG	NA	NA
	2446859-65-C/G	AP	Zm00001d042777	IG	A	Basic leucine zipper protein%3B Liguleless2	DOG1
	4584446-12-G/C	NH	Zm00001d043494	IG	A	elongation factor family protein	TypA_BipA superfamily|| TypA superfamily
	4583173-13-T/C	GWL	Zm00001d044409	-1,195	A	MYB-related transcription factor	myb_SHAQKYF
			Zm00001d044410	IG	A	Cycloartenol synthase	F-box-like
**4**	2381322-13-C/G	FAW	Zm00001d049175	15,726	B	OSJNBaNANA43A12.2NA protein	NA
	4779016-24-C/T	NH	Zm00001d049295	IG	A	Auxin response factor 2	Auxin_resp || B3|| AUX_IAA superfamily
	4577027-47-G/A	GWL	Zm00001d049854	-672	A	3'-5'-exoribonuclease family protein	RNase_PH superfamily
	**100220678-45-A/G**	**FAW-FP**	**Zm00001d050286**	**789**	**C**	**Sphingolipid delta(4)-desaturase DES1-like**	PLN2579 superfamily
	4771330-29-T/C	NH	Zm00001d052111	IG	A	DENN (AEX-3) domain-containing protein	DENN || uDENN
	2619648-16-T/C	GWL	Zm00001d052377	IG	A	Pentatricopeptide repeat protein PPR868-14 isoform 1%3B	PLN381 superfamily
**5**	4589321-22-G/A	AK	Zm00001d013314	IG	A	Vps51/Vps67 family (components of vesicular transport) protein	Vps51 || COG2 superfamily
	7048960-37-T/G	NH	Zm00001d014099	IG	A	Disease resistance protein RPM1	NB-ARC superfamily || RX-CC_like superfamily || LRR || HHH_5 superfamily
	7049219-26-T/C	FAW	Zm00001d015956	IG	A	NAD(P)-binding Rossmann-fold superfamily protein	PLN2662
	4584182-35-C/G	FAW	Zm00001d016271	IG	B	NA	DUF761
			Zm00001d016272	2,578	NGI	NA	NA
	4774140-50-G/A	FP	Zm00001d017703	-2,370	B	survival motor neuron protein	NA
			Zm00001d017704	IG	A	Target of Myb protein 1	VHS || GAT_GGA_like_plant

Chr.	QTNs	Trait	Candidate Gene	DQTN	CGC	Gene description	Conserved protein domain
**6**	4587005-7-C/G	AK-NH	NA	NA	NCG	NA	NA
	4771590-67-A/T	FP	Zm00001d036215	IG	A	MAP kinase7	PKc_like superfamily
	5586936-13-T/C	FP	Zm00001d036830	IG	A	Putative calcium-dependent lipid-binding (CaLB domain) family protein	C2_ArfGAP ǁ ArfGapM-associated superfamily ǁ ArfGap superfamilyǁ DUF48 superfamily
	4579331-18-T/C	AP	NA	NA	NCG	NA	NA
	4764931-6-G/A	FP-AP	Zm00001d039049	IG	A	Putative homeodomain-like transcription factor superfamily protein%3B SANT/MYB protein	SANT
			Zm00001d039050	1,571	A	DNA-3-methyladenine glycosylase 1	AlkA
			Zm00001d039048	-2,212	NGI	NA	NA
**7**	4771072-39-A/G	GWL	Zm00001d018807	81	A	Leucine-rich repeat receptor-like serine/threonine-protein kinase	PLN113 superfamily
	5587204-51-A/C	AK	Zm00001d001255	-253	B	NA	NA
	4580355-27-G/A	GWL-AP	Zm00001d022267	IG	A	chromatin remodeling factor18	DEXHc_HARP_SMARCAL1 ǁ HepA
**8**	4773640-63-T/A	FP	Zm00001d008175	-1,289	C	S-adenosylmethionine synthase 1	PLN2243 superfamily
			Zm00001d008176	IG	A	Squamosa promoter-binding protein-like (SBP domain) transcription factor family protein	SBP
	4770550-8-G/C	GWL	Zm00001d008669	-96	A	Histone H4	PLN35
	2504966-32-A/G	FAW	*Zm00001d010095*	3,287	NGI	NA	NA
			Zm00001d010094	IG	B	Dehydrin family protein expressed ǁ Embryogenic-cell protein 4NA (Ecp4NA)	Dehydrin
	**2559495-18-T/G**	**FAW-AK**	**Zm00001d011308**	**IG**	**A**	**Ubiquitin-like superfamily protein**	Ubl_SUMO_like
	2610943-54-T/C	GWL-AP	*Zm00001d012218*	-1,236	NGI	NA	NA
			Zm00001d012219	1,885	A	NA	F-box-like

Chr.	QTNs	Trait	Candidate Gene	DQTN	CGC	Gene description	Conserved protein domain
	2376195-62-T/G	FP	Zm00001d012553	IG	A	octopine synthase binding factor4	DOG1|| bZIP_HBP1b-like
	4579847-66-T/G	FP	Zm00001d012761	IG	A	Protein kinase family protein	STKc_CK1
**9**	4771587-19-T/C	AK	Zm00001d046069	-5,859	B	NA	FANCI_S4 superfamily
			Zm00001d027105	IG	C	NA	NA
			Zm00001d046070	-10,149	NGI	NA	NA
	100023814-29-T/G	AK-FP	Zm00001d047162	IG	A	UDP-Glycosyltransferase superfamily protein	NA
	**9682691-38-C/T**	**FP-FAW**	**Zm00001d047412**	**IG**	**A**	**Protein phosphatase 2C 32**	PP2Cc
	4764675-42-C/G	AP	Zm00001d047518	IG	A	Nardilysin-like	Ptr superfamily
**10**	4582917-12-A/G	GWL	Zm00001d024816	IG	A	chromatin complex subunit A	PLN3142 superfamily
	2539012-9-A/C	GWL-FP-AP	Zm00001d025013	IG	NGI	NA	NA
			Zm00001d025014	2,385	NGI	NA	NA
	100298755-56-T/C	FAW	Zm00001d025153	IG	A	Phospholipid-transporting ATPase 2	HAD_like superfamily || PhoLip_ATPase_C
	4776702-53-G/A	AK	NA	NA	NGC	NA	NA
	7061499-37-A/G	AP	Zm00001d026042	971	A	Probable purine permease 11	PUNUT superfamily

Chr=Chromosome; DQTN=Distance from QTN, a negative (-) distance means the CG is upstream the QTN and a positive value indicates the CG located downstream the QTN; IG=Inside gene; NA=Not available; NCG=No candidate gene; NGI=No genetic information. In bold are QTNs and CGs for combined fall armyworm (FAW) and maize weevil (MW) traits such as Grain weight loss (GWL), emerged Adult progenies (AP), Flour produced (FP), number of Affected kernels (AK), and Number of grain holes (NH).

Table S3. 107 Network-CGs with their chromosome (Chr), start and end position based on the AGPv4 maize genome reference genome and descriptions.

Gene stable ID	Chr	Start (bp)	End (bp)	Gene name	Gene description
Zm00001d027760	1	13,023,613	13,024,482	NA	Histone H2A
Zm00001d029075	1	57,258,183	57,259,499	NA	CBL-interacting serine/threonine-protein kinase 10
Zm00001d029263	1	64,042,656	64,045,687	NA	Chaperonin
Zm00001d033746	1	272,697,870	272,704,606	phosphoglucomutase1	phosphoglucomutase1
Zm00001d033822	1	274,809,375	274,810,292	NA	Probable histone H2AXa
Zm00001d034089	1	283,693,163	283,695,688	NA	Probable receptor-like protein kinase
Zm00001d034372	1	291,107,391	291,111,360	NA	Calcium-dependent protein kinase 1
Zm00001d034562	1	296,437,168	296,441,398	NA	Calcium-dependent protein kinase 2
Zm00001d034663	1	299,194,181	299,196,764	alpha-expansin4	alpha-expansin4

Gene stable ID	Chr	Start (bp)	End (bp)	Gene name	Gene description
Zm00001d034671	1	299,442,286	299,444,334	NA	Lectin-like receptor kinase 7
Zm00001d002172	2	7,110,526	7,119,435	NA	G-type lectin S-receptor-like serine/threonine-protein kinase B120
Zm00001d002172	2	7,110,526	7,119,435	NA	G-type lectin S-receptor-like serine/threonine-protein kinase B120
Zm00001d002253	2	8,988,852	8,989,265	NA	60S ribosomal protein L27
Zm00001d003019	2	29,627,526	29,635,338	NA	Protein kinase superfamily protein
Zm00001d003673	2	53,484,601	53,488,150	NA	Protein kinase superfamily protein
Zm00001d003725	2	56,544,752	56,545,162	H3C2	Histone H3.2
Zm00001d003730	2	56,683,255	56,683,907	H3C2	Histone H3.2
Zm00001d005808	2	189,439,983	189,444,202	NA	Probable ethanolamine kinase
Zm00001d005964	2	194,057,868	194,060,798	bHLH-transcription factor 151	Transcription factor bHLH76
Zm00001d006008	2	195,179,025	195,182,945	NA	Heat shock protein 90-2
Zm00001d006536	2	210,665,909	210,668,852	NA	Cysteine-rich receptor-like protein kinase 10
Zm00001d038708	2	163,090,769	163,092,715	NA	Proline-rich receptor-like protein kinase PERK15
Zm00001d007192	2	224,425,013	224,429,818	NA	T-complex protein 1 subunit zeta
Zm00001d007166	2	223,634,679	223,635,971	NA	CBL-interacting serine/threonine-protein kinase 4
Zm00001d007167	2	223,667,838	223,669,229	NA	CBL-interacting serine/threonine-protein kinase 15
Zm00001d041215	3	105,826,354	105,830,061	NA	ATP binding protein
Zm00001d040996	3	89,082,044	89,084,928	NA	Calcium-dependent protein kinase 1
Zm00001d045359	3	19,532,789	19,536,828	NA	Mitogen-activated protein kinase kinase 2
Zm00001d042475	3	168,871,221	168,878,750	NA	Probable thimet oligopeptidase
Zm00001d043480	3	201,473,205	201,476,633	NA	Proline-rich receptor-like protein kinase PERK15
Zm00001d043841	3	211,598,479	211,613,691	NA	Katanin p60 ATPase-containing subunit A1
Zm00001d043955	3	214,775,698	214,777,826	NA	Eukaryotic translation initiation factor 3 subunit D
Zm00001d043923	3	213,970,968	213,973,403	NA	PAN domain-containing protein
Zm00001d044246	3	222,967,858	222,968,792	NA	Histone H2A
Zm00001d044301	3	224,583,950	224,589,560	protein phosphatase homolog13	Protein phosphatase 2C ABI2
Zm00001d044639	3	233,811,186	233,813,372	NA	L-type lectin-domain containing receptor kinase IX.1
Zm00001d049286	4	24,420,976	24,426,546	NA	LRR receptor-like serine/threonine-protein kinase EFR

Gene stable ID	Chr	Start (bp)	End (bp)	Gene name	Gene description
Zm00001d052340	4	187,401,028	187,402,359	NA	CBL-interacting serine/threonine-protein kinase 10
Zm00001d000110	4	187,964	193,349	MYB-related-transcription factor 61	SWI/SNF complex subunit SWI3C
Zm00001d053087	4	213,023,215	213,025,176	NA	G-type lectin S-receptor-like serine/threonine-protein kinase SD2-5
Zm00001d053135	4	215,474,432	215,476,369	NA	D-mannose binding lectin family protein
Zm00001d013428	5	11,195,010	11,201,017	phosphoglucomutase2	phosphoglucomutase2
Zm00001d014152	5	34,251,816	34,258,225	NA	Cationic amino acid transporter 4 vacuolar
Zm00001d014291	5	39,715,418	39,722,456	NA	Probable protein phosphatase 2C 71
Zm00001d016381	5	160,203,687	160,206,309	NA	Histone deacetylase
Zm00001d017069	5	184,380,955	184,383,871	NA	Inorganic phosphate transporter 2-1 chloroplastic
Zm00001d035476	6	28,516,750	28,525,006	NA	L-type lectin-domain containing receptor kinase VIII.1
Zm00001d035588	6	35,148,537	35,151,188	NA	Serine/threonine-protein kinase
Zm00001d035747	6	44,780,803	44,789,355	argonaute1a	argonaute1a
Zm00001d036097	6	72,141,933	72,145,178	NA	Putative DUF26-domain receptor-like protein kinase family protein
Zm00001d036322	6	84,112,004	84,117,155	NA	Ribonucleoside-diphosphate reductase
Zm00001d036879	6	105,515,248	105,521,341	NA	Putative CBL-interacting protein kinase family protein
Zm00001d036917	6	106,360,618	106,367,670	argonaute1NAb	argonaute10b
Zm00001d036986	6	108,327,528	108,338,061	NA	ABC transporter G family member 29
Zm00001d038282	6	153,243,817	153,250,372	NA	Putative LSTK-1-like/NimA-related protein kinase family protein isoform; 3B Putative LSTK-1-like/NimA-related protein kinase family protein isoform 2
Zm00001d038409	6	156,603,002	156,614,078	NA	Calcium-dependent protein kinase 13
Zm00001d038708	2	163,090,769	163,092,715	NA	Proline-rich receptor-like protein kinase PERK15
Zm00001d019042	7	13,942,239	13,944,164	NA	Actin-related protein 2/3 complex subunit 3
Zm00001d019045	7	14,093,201	14,093,758	NA	Histone H2A
Zm00001d019084	7	15,693,408	15,695,533	NA	RNA-binding (RRM/RBD/RNP motifs) family protein
Zm00001d020134	7	94,949,585	94,962,130	NA	ABC transporter G family member 40
Zm00001d020138	7	95,424,853	95,428,598	NA	L-type lectin-domain containing receptor kinase IX.1
Zm00001d020396	7	111,240,634	111,244,556	trehalose-6-phosphate synthase13	trehalose-6-phosphate synthase13

Gene stable ID	Chr	Start (bp)	End (bp)	Gene name	Gene description
Zm00001d020496	7	119,111,856	119,113,250	NA	CBL-interacting serine/threonine-protein kinase 5
Zm00001d020497	7	119,140,649	119,141,980	NA	CBL-interacting serine/threonine-protein kinase 6
Zm00001d020584	7	123,703,834	123,704,145	H4C7	Histone H4
Zm00001d020585	7	123,712,201	123,712,512	H4C7	Histone H4
Zm00001d021139	7	143,856,218	143,860,073	NA	Calcium-dependent protein kinase 24
Zm00001d021255	7	147,034,121	147,042,045	NA	NA
Zm00001d021300	7	148,227,448	148,227,942	NA	Histone H2A
Zm00001d021434	7	152,277,267	152,279,488	NA	G-type lectin S-receptor-like serine/threonine-protein kinase B120
Zm00001d021434	7	152,277,267	152,279,488	NA	G-type lectin S-receptor-like serine/threonine-protein kinase B120
Zm00001d021477	7	153,335,991	153,336,302	H4C7	Histone H4
Zm00001d022307	7	174,845,532	174,847,522	NA	10 kDa chaperonin
Zm00001d022547	7	179,784,948	179,788,293	NA	CBL-interacting serine/threonine-protein kinase 3
Zm00001d008468	8	9,622,565	9,639,429	NA	PR5-like receptor kinase
Zm00001d008477	8	9,807,195	9,830,853	receptor-like kinase4	receptor-like kinase4
Zm00001d008581	8	13,548,214	13,555,561	NA	LEAF RUST 10 DISEASE-RESISTANCE LOCUS RECEPTOR-LIKE PROTEIN KINASE-like 1.1
Zm00001d010459	8	116,264,102	116,265,454	NA	Putative CBL-interacting protein kinase family protein
Zm00001d010529	8	119,419,987	119,423,027	NA	Probable mediator of RNA polymerase II transcription subunit 37c
Zm00001d010461	8	116,331,820	116,334,299	NA	Sm-like protein LSM5
Zm00001d010575	8	120,733,570	120,733,881	H4C7	Histone H4
Zm00001d010659	8	123,186,799	123,189,450	NA	Putative calcium-dependent protein kinase family protein
Zm00001d010743	8	126,502,112	126,503,641	NA	Putative CBL-interacting protein kinase family protein
Zm00001d011392	8	149,387,614	149,392,198	NA	Calcium-dependent protein kinase%2C isoform 2%3B Putative calcium-dependent protein kinase family protein isoform 1%3B Putative calcium-dependent protein kinase family protein isoform 2
Zm00001d011628	8	156,686,200	156,694,580	NA	PR5-like receptor kinase
Zm00001d045190	9	15,650,398	15,654,451	NA	Putative WAK family receptor-like protein kinase
Zm00001d045192	9	15,720,802	15,728,927	hybrid proline-rich protein1	Ribonucleoside-diphosphate reductase large subunit
Zm00001d045359	3	19,532,789	19,536,828	NA	Mitogen-activated protein kinase kinase 2

Gene stable ID	Chr	Start (bp)	End (bp)	Gene name	Gene description
Zm00001d045839	9	42,917,485	42,919,506	NA	Putative lectin-like receptor protein kinase family protein
Zm00001d045838	9	42,913,838	42,915,841	NA	Putative lectin-like receptor protein kinase family protein
Zm00001d046438	9	89,734,202	89,740,800	argonaute1NA1	argonaute101
Zm00001d048460	9	156,719,295	156,724,276	NA	CBL-interacting serine/threonine-protein kinase 9
Zm00001d047531	9	134,014,122	134,018,935	NA	Putative AGC-like protein kinase family protein isoform; 3B Putative AGC-like protein kinase family protein isoform; 3B Putative AGC-like protein kinase family protein isoform; 3B Serine/threonine-protein kinase AtPK19
Zm00001d047758	9	141,026,557	141,029,745	MAP kinase1	MAP kinase1
Zm00001d047858	9	143,631,572	143,633,228	NA	Protein STRICTOSIDINE SYNTHASE-LIKE 13
Zm00001d047909	9	144,963,925	144,969,440	NA	transcription activators;DNA binding;RNA polymerase II transcription factors;catalytics;transcription initiation factors
Zm00001d048203	9	152,377,111	152,382,143	NA	Probable protein phosphatase 2C BIPP2C1
Zm00001d024591	10	79,578,452	79,580,065	NA	SnRNP core Sm protein Sm-X5-like protein
Zm00001d024637	10	81,459,381	81,466,000	NA	L-type lectin-domain containing receptor kinase V.9
Zm00001d024637	10	81,459,381	81,466,000	NA	L-type lectin-domain containing receptor kinase V.9
Zm00001d024903	10	93,908,317	93,911,235	heat shock protein, 9NA kDa	heat shock protein 90 kDa
Zm00001d025406	10	117,441,376	117,441,837	H3C2	Histone H3.2
Zm00001d025913	10	133,665,354	133,665,806	NA	Histone H2B
Zm00001d025920	10	133,768,161	133,770,170	NA	Putative lectin-like receptor protein kinase family protein
Zm00001d025997	10	135,684,027	135,687,837	NA	Protein kinase superfamily protein
Zm00001d026489	10	146,944,932	146,946,110	NA	OSJNBb0022F16.11 protein; protein

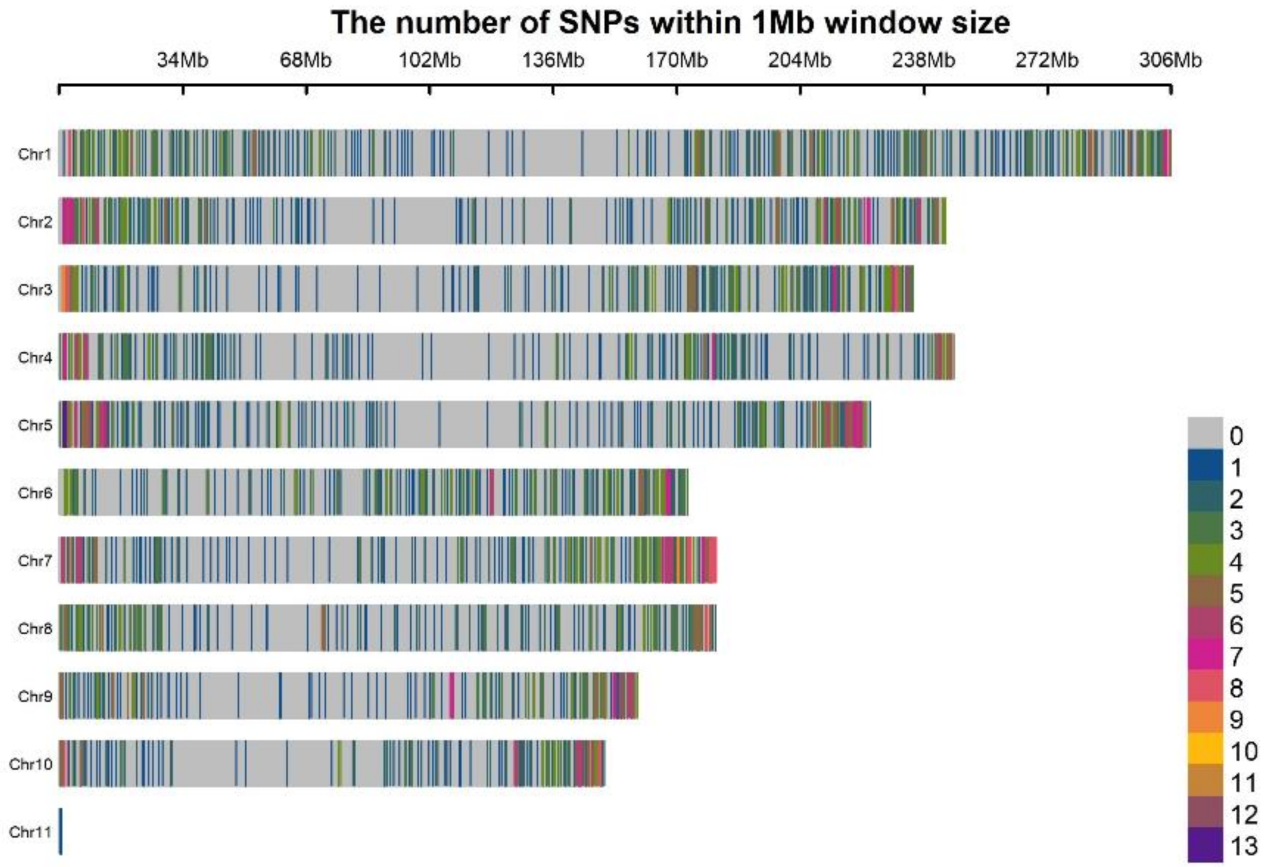

Figure S1. Distribution of the 3124 LDPSNPs across the 10 maize chromosomes. Chr11 groups SNPs which were not mapped in any chromosomes.

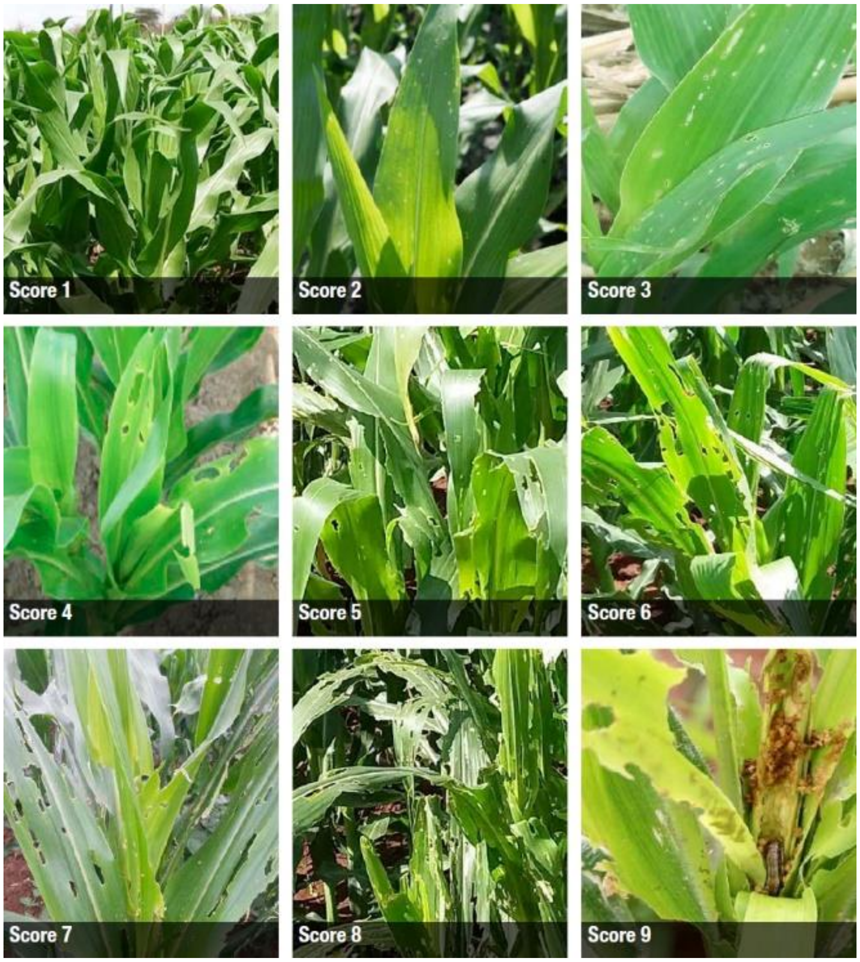

Figure S2. Rating of maize plants based on foliar damage by FAW (45)**.**

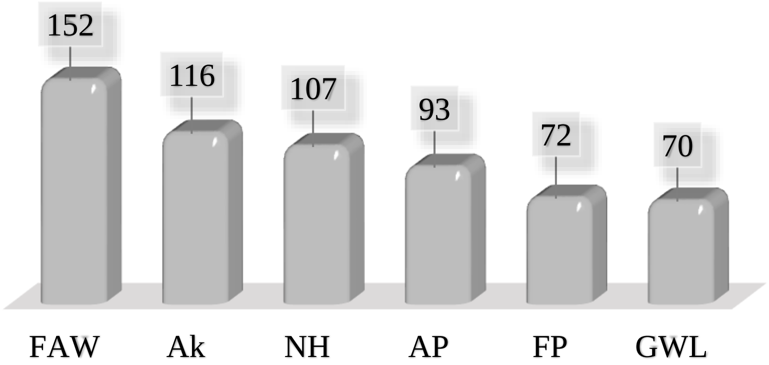

Figure S3. Number of principal components (PC) included in the GWAS model for fall armyworm (FAW) damage, and for the different maize weevil (MW) resistance traits: number of affected kernels (AK), number grain holes (NH), number of emerger adult progenies (AP), total amount of flour produced (FP), and grain weight loss (GWL).

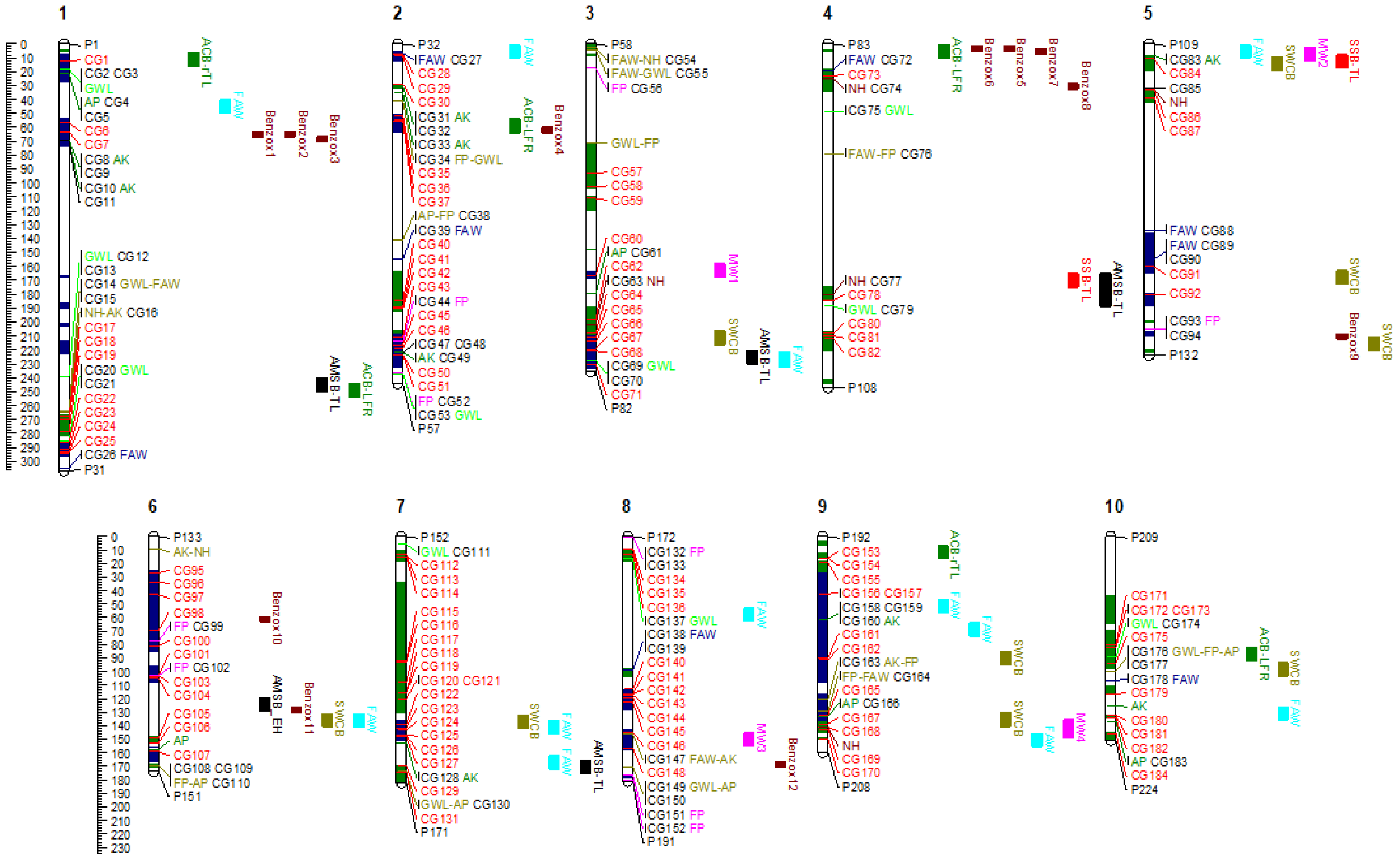

Figure S4. Physical map based on the AGPv4 maize reference genome showing on the chromosomes the single (in green) and multiple (in blue) insect resistance genomic regions (IRGR) and on the side their colocalizations with QTL for Asian corn borer (ACB), southwestern corn borer (SWCB), Fall armyworm (FAW), Maize weevil (MW), African maize stalk borer (AMSB), spotted SB (SSB) resistance, and maize benzoxazinoids (Benzox) content. Start and end positions of the chromosomes (P1 to 224) and the location of the GWAS-CGs (in blue) and Network-CGs (in red) associated with maize resistance to FAW and MW were placed on the chromosomal bars. TL=Tunnel length, rTL=Relative TL, LFR=leaf feeding rate, EH=Exit holes.

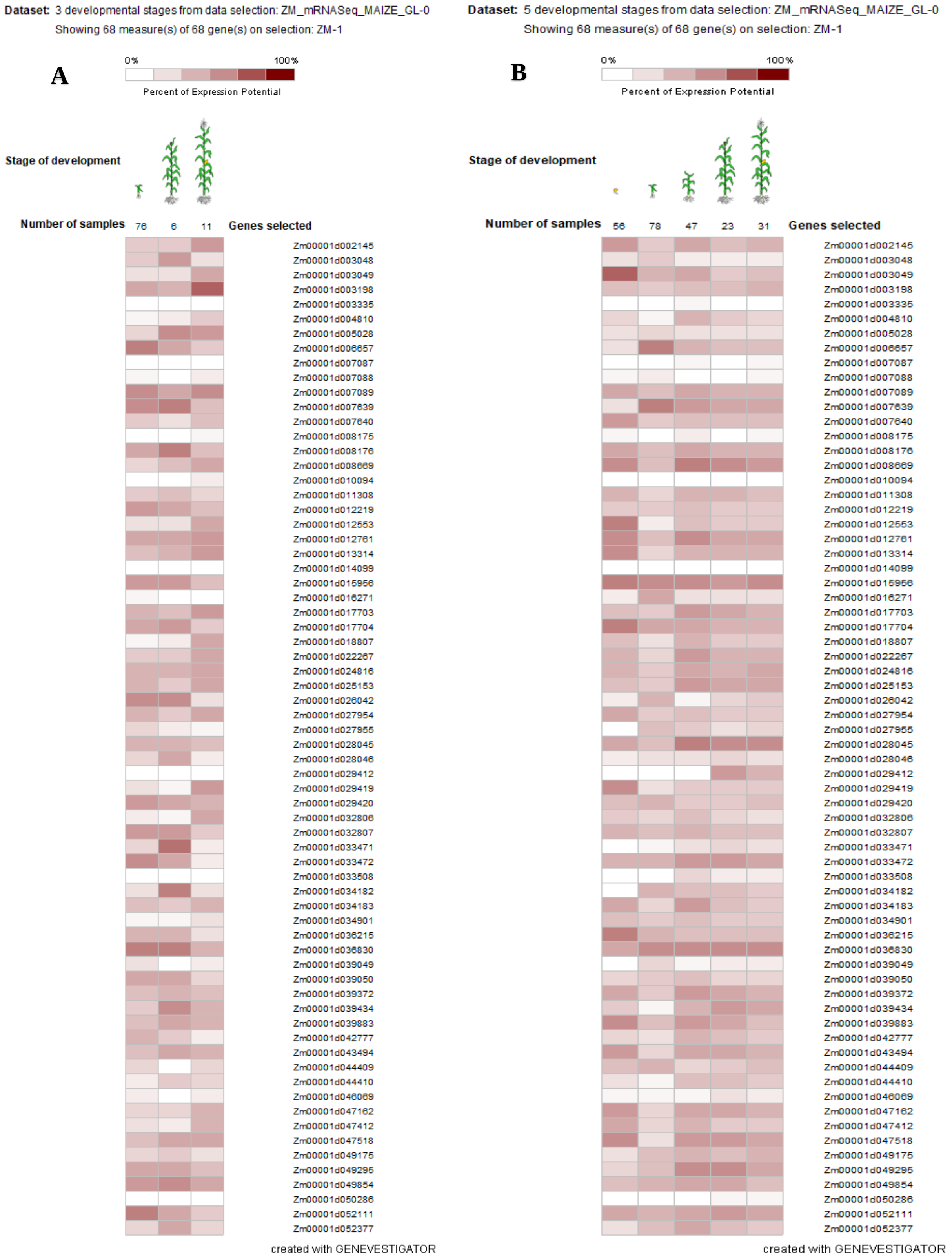

Figure S5. ***In-silico*** **expression profile of the CG at different maize developmental stages relevant to FAW and MW damage under different biotic (A) and abiotic (B) stress conditions.**

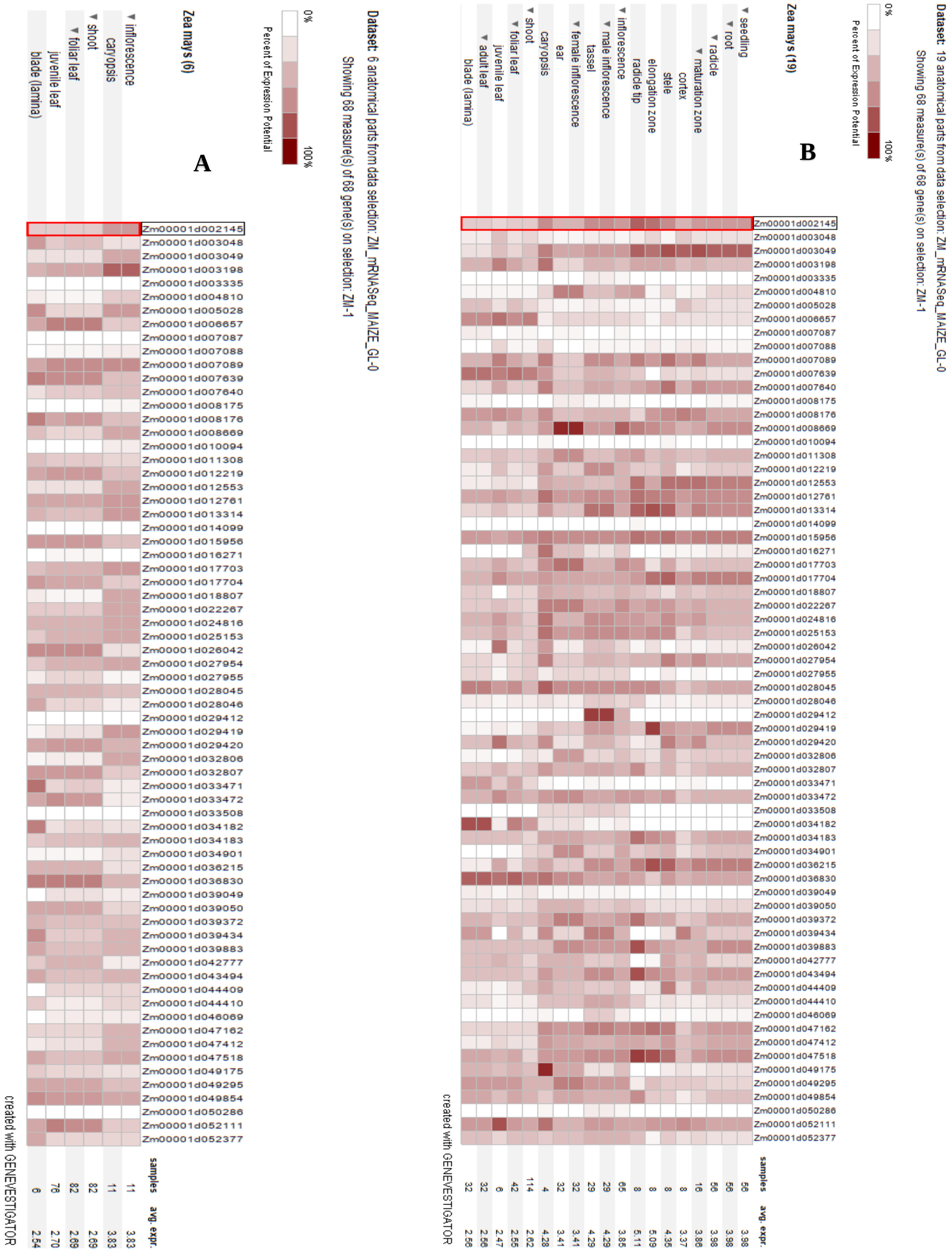

Figure S6. ***In-silico*** **expression profile of the CG in different maize organs relevant to FAW and MW damage under different biotic (A) and abiotic (B) stress conditions.**

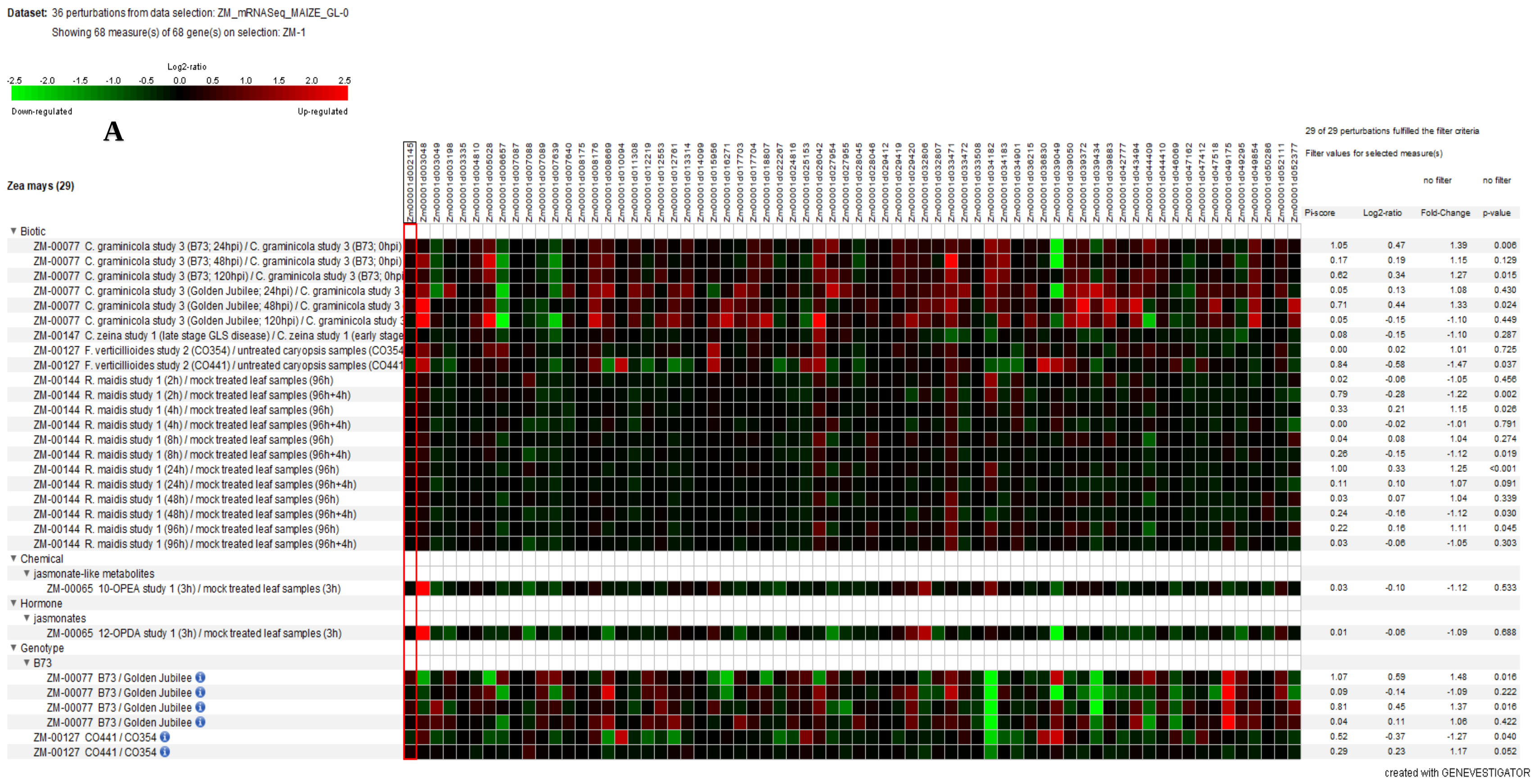
Dataset: 36 perturbations from data selection: ZM_mRNASeq_MAIZE_GL-0
Showing 68 measure(s) of 68 gene(s) on selection: ZM-1
Log2-ratio
-2.5 -2.0 -1.5 -1.0 -0.5 0.0 0.5 1.0 1.5 2.0 2.5
Down-regulated
Up-regulated
A
Zea mays (29)
29 of 29 perturbations fulfilled the filter criteria
Filter values for selected measure(s)
no filter
no filter
Pi-score Log2-ratio Fold-Change p-value
Biotic
ZM-00077 C. graminicola study 3 (B73; 24hpi) / C. graminicola study 3 (B73; 0hpi) 1.05 0.47 1.39 0.006
ZM-00077 C. graminicola study 3 (B73; 48hpi) / C. graminicola study 3 (B73; 0hpi) 0.17 0.19 1.15 0.129
ZM-00077 C. graminicola study 3 (B73; 120hpi) / C. graminicola study 3 (B73; 0hpi 0.62 0.34 1.27 0.015
ZM-00077 C. graminicola study 3 (Golden Jubilee; 24hpi) / C. graminicola study 3 0.05 0.13 1.08 0.430
ZM-00077 C. graminicola study 3 (Golden Jubilee; 48hpi) / C. graminicola study 3 0.71 0.44 1.33 0.024
ZM-00077 C. graminicola study 3 (Golden Jubilee; 120hpi) / C. graminicola study 3 0.05 -0.15 -1.10 0.449
ZM-00147 C. zeina study 1 (late stage GLS disease) / C. zeina study 1 (early stage 0.08 -0.15 -1.10 0.287
ZM-00127 F. verticillioides study 2 (CO354) / untreated caryopsis samples (CO354 0.00 0.02 1.01 0.725
ZM-00127 F. verticillioides study 2 (CO441) / untreated caryopsis samples (CO441 0.84 -0.58 -1.47 0.037
ZM-00144 R. maidis study 1 (2h) / mock treated leaf samples (96h) 0.02 -0.06 -1.05 0.456
ZM-00144 R. maidis study 1 (2h) / mock treated leaf samples (96h+4h) 0.79 -0.28 -1.22 0.002
ZM-00144 R. maidis study 1 (4h) / mock treated leaf samples (96h) 0.33 0.21 1.15 0.026
ZM-00144 R. maidis study 1 (4h) / mock treated leaf samples (96h+4h) 0.00 -0.02 -1.01 0.791
ZM-00144 R. maidis study 1 (8h) / mock treated leaf samples (96h) 0.04 0.08 1.04 0.274
ZM-00144 R. maidis study 1 (8h) / mock treated leaf samples (96h+4h) 0.26 -0.15 -1.12 0.019
ZM-00144 R. maidis study 1 (24h) / mock treated leaf samples (96h) 1.00 0.33 1.25 <0.001
ZM-00144 R. maidis study 1 (24h) / mock treated leaf samples (96h+4h) 0.11 0.10 1.07 0.091
ZM-00144 R. maidis study 1 (48h) / mock treated leaf samples (96h) 0.03 0.07 1.04 0.339
ZM-00144 R. maidis study 1 (48h) / mock treated leaf samples (96h+4h) 0.24 -0.16 -1.12 0.030
ZM-00144 R. maidis study 1 (96h) / mock treated leaf samples (96h) 0.22 0.16 1.11 0.045
ZM-00144 R. maidis study 1 (96h) / mock treated leaf samples (96h+4h) 0.03 -0.06 -1.05 0.303
Chemical
jasmonate-like metabolites
ZM-00065 10-OPEA study 1 (3h) / mock treated leaf samples (3h) 0.03 -0.10 -1.12 0.533
Hormone
jasmonates
ZM-00065 12-OPDA study 1 (3h) / mock treated leaf samples (3h) 0.01 -0.06 -1.09 0.688
Genotype
B73
ZM-00077 B73 / Golden Jubilee 1.07 0.59 1.48 0.016
ZM-00077 B73 / Golden Jubilee 0.09 -0.14 -1.09 0.222
ZM-00077 B73 / Golden Jubilee 0.81 0.45 1.37 0.016
ZM-00077 B73 / Golden Jubilee 0.04 0.11 1.06 0.422
ZM-00127 CO441 / CO354 0.52 -0.37 -1.27 0.040
ZM-00127 CO441 / CO354 0.29 0.23 1.17 0.052
created with GENEVESTIGATOR

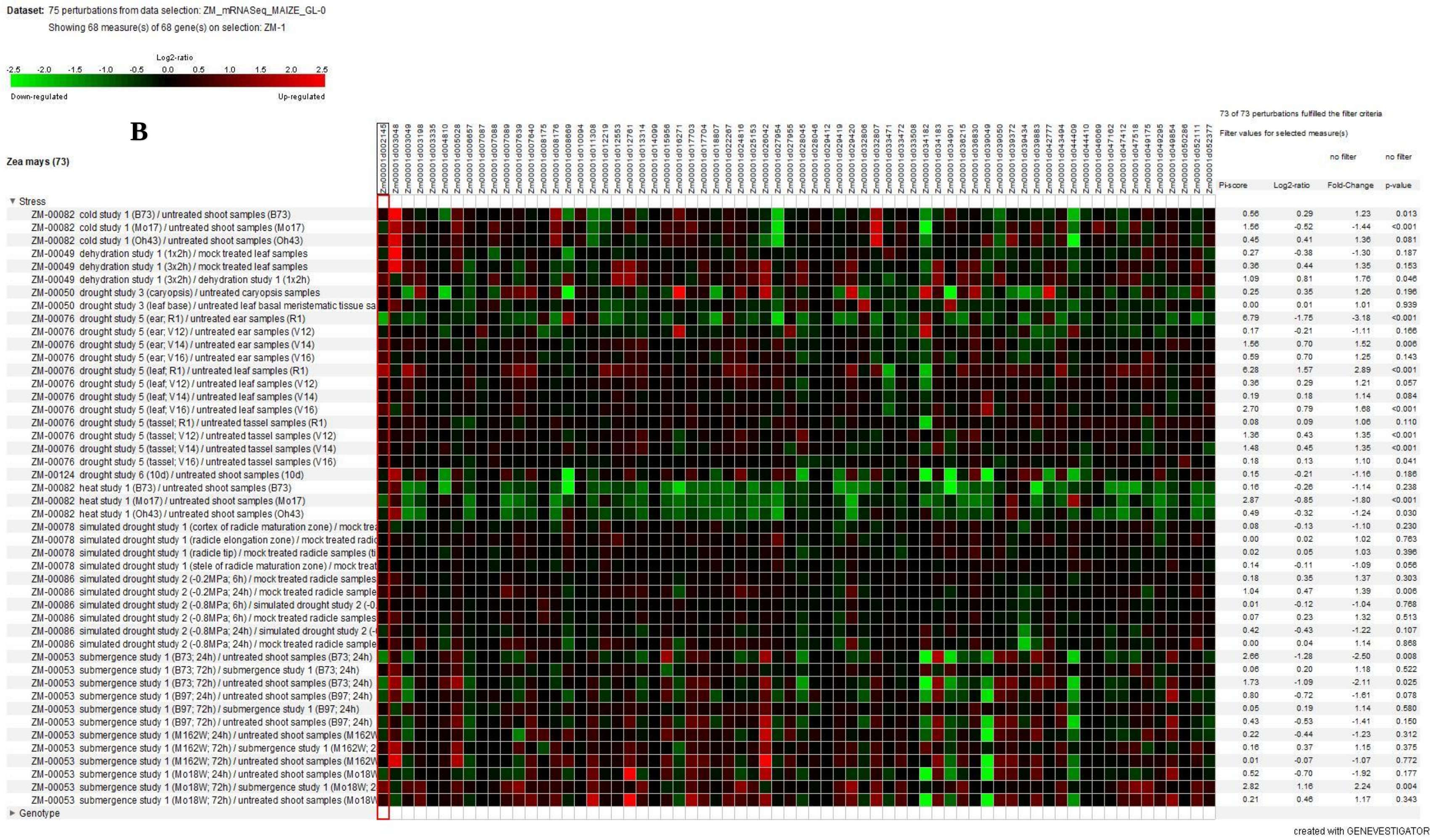

Figure S7. ***In-silico*** **expression profile of the CG under different biotic stress, jamonates and jasmonate-like treatments (A) and abiotic (B) stress conditions.**

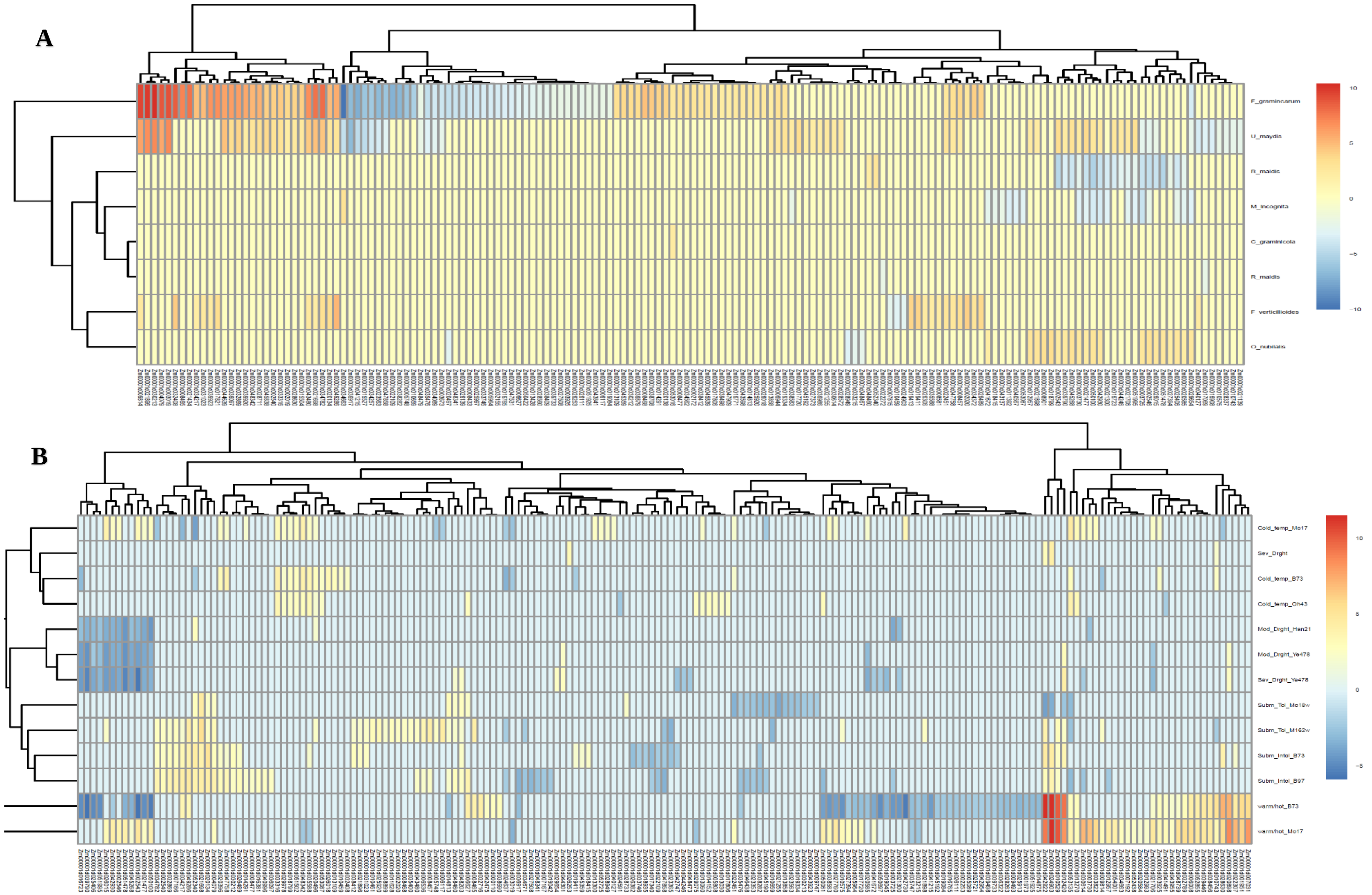

Figure S8. Network-based candidate genes differentially expressed under different biotic agents (A) including *Fusarium graminearum* and *verticelloides*, *Meloidogyne incognita*, *Ostrinia nubilalis*, *Rhopalosiphum maidis* and *Ustilago maydis*, and under different abiotic stress conditions (B) such as cold temperature, drought, heat, and submergence.

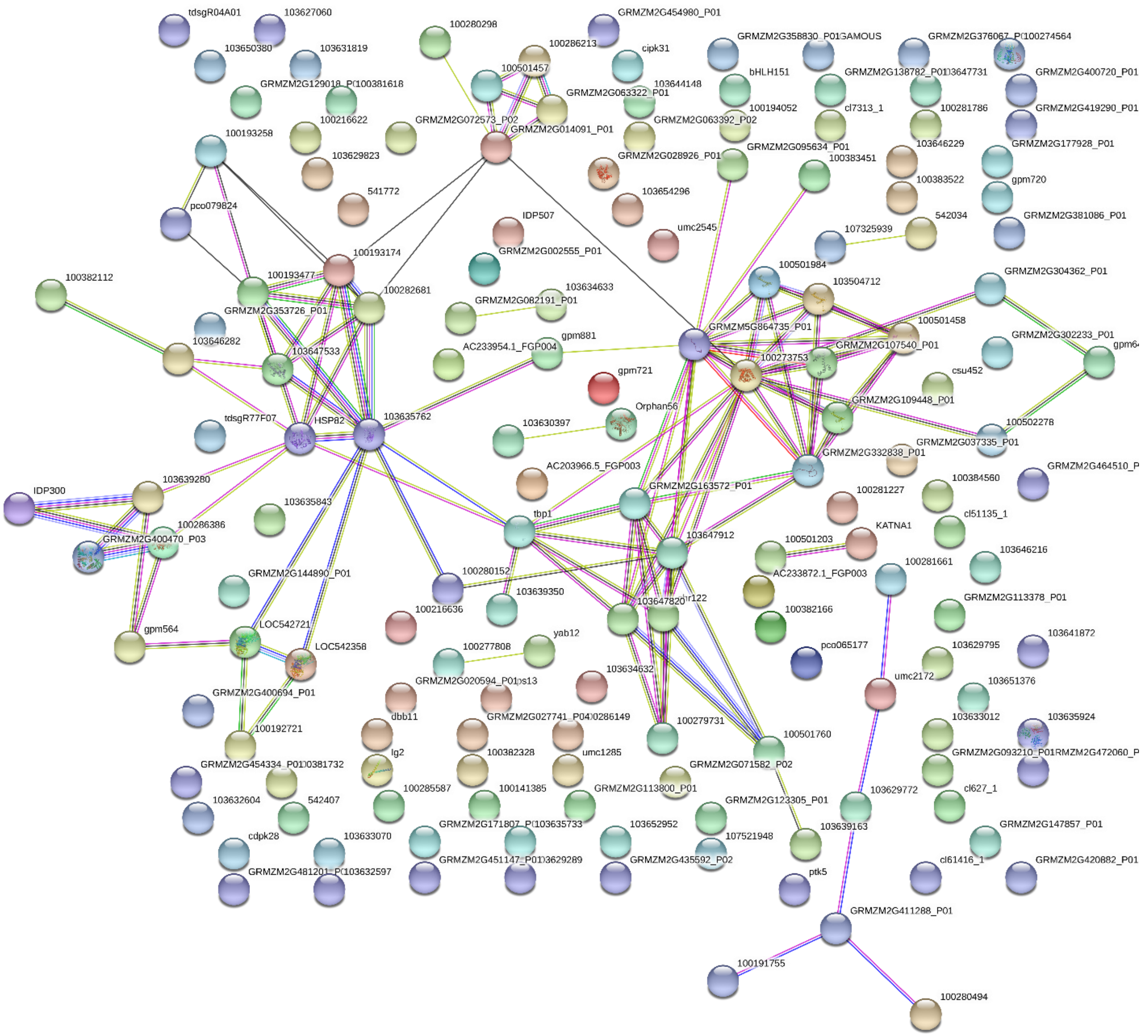

Figure S9. Protein-protein interaction network (edges) linking the CGs (nodes).
Names of the proteins (dots) of the protein-protein interaction network are based on the STRING protein ids.
